# ﻿Disintegration of the genus *Prosopis* L. (Leguminosae, Caesalpinioideae, mimosoid clade)

**DOI:** 10.3897/phytokeys.205.75379

**Published:** 2022-08-22

**Authors:** Colin E. Hughes, Jens J. Ringelberg, Gwilym P. Lewis, Santiago A. Catalano

**Affiliations:** 1 Department of Systematic & Evolutionary Botany, University of Zurich, Zollikerstrasse 107, 8008 Zurich, Switzerland University of Zürich Zurich Switzerland; 2 Accelerated Taxonomy Department, Royal Botanic Gardens, Kew, Richmond, Surrey, TW9 3AE, UK University of Zurich Zurich Switzerland; 3 Unidad Ejecutora Lillo, Consejo Nacional de Investigaciones Científicas y Técnicas – Fundación Miguel Lillo, Miguel Lillo 251, 4000 S. M. de Tucumán, Argentina Accelerated Taxonomy Department, Royal Botanic Gardens Richmond United Kingdom; 4 Facultad de Ciencias Naturales e Instituto Miguel Lillo, Universidad Nacional de Tucumán, Miguel Lillo 205, 4000 S. M. de Tucumán, Argentina Consejo Nacional de Investigaciones Cientificas y Tecnicas, Fundacion Miguel Lillo S.M. de Tucuman Argentina

**Keywords:** *Anonychium*, Fabaceae, generic delimitation, *Indopiptadenia*, monophyly, *Neltuma*, *Strombocarpa*, taxonomy, *Xerocladia*

## Abstract

Robust evidence from phylogenomic analyses of 997 nuclear genes has recently shown, beyond doubt, that the genus *Prosopis* is polyphyletic with three separate lineages, each with affinities to other genera of mimosoids: (i) *Prosopisafricana* is an isolated lineage placed in the grade of *Plathymenia*, *Newtonia* and *Fillaeopsis* that subtends the core mimosoid clade; (ii) the remaining Old World species of *Prosopis* form a clade that is sister to the Indo-Nepalese monospecific genus *Indopiptadenia* and (iii) New World *Prosopis* has the Namibian / Namaqualand monospecific endemic genus *Xerocladia* nested within it. This means that it is now clear that maintaining the unity of the genus *Prosopis* sensu Burkart (1976) is no longer tenable. These three distinct lineages of *Prosopis* species correspond directly to Burkart’s (1976) sectional classification of the genus, to previously recognised genera and to the differences in types of armature that underpin Burkart’s sections. Here, we address this non-monophyly by resurrecting three segregate genera – *Anonychium*, *Neltuma* and *Strombocarpa* and provide 57 new name combinations where necessary, while maintaining the morphologically distinctive and geographically isolated genera *Xerocladia* and *Indopiptadenia*. The genus *Prosopis* itself is reduced to just three species and an emended description is presented. The impacts of these name changes for a genus of such high ecological and human use importance are discussed. These impacts are mitigated by clear differences in armature which facilitate identification and by potential benefits from the deeper biological understanding brought about by recognition of these divergent lineages at generic rank. We provide an identification key to genera and present a map showing the distributions of the segregate genera, as well as drawings and photos illustrating variation in armature and fruits.

## ﻿Introduction

Burkart’s (1976) worldwide taxonomic monograph of the genus *Prosopis* L. recognised 44 species placed in five sections. Since then, 13 additional species have been described (Schinini 1981; Earl and Lux 1991; Palacios 2006; Vásquez Núñez et al. 2009; De Mera et al. 2019) one of which, *P.bonplanda* P.R. Earl & Lux, was subsequently treated as a synonym by Palacios (2006). All of these additional species belong morphologically in section Algarobia DC., such that the generic unity and infrageneric classification, proposed by Burkart (1976), remain the current framework for understanding the genus. Following Bentham (1875), Burkart (1976) justified the generic unity of a widely delimited *Prosopis*, based on the broad uniformity of flowers and fruits across *Prosopis* s.l. Perhaps the most important uniting feature was the modified indehiscent cylindrical or thickened legume, with a more or less sugary, fleshy or fibrous mesocarp and an endocarp more or less hardened and segmented into one-seeded coriaceous to bony seed chambers, these closed or sometimes opening easily. Fruits of this type are eagerly consumed by herbivores, including all kinds of livestock, the seeds benefiting from scarification as they pass through the digestive tract and as a result being widely dispersed (see below), a seed dispersal syndrome that unites all species of *Prosopis* s.l. Moreover, Burkart (1976) explicitly downplayed vegetative characters and notably variation in armature, as of less significance for classification, stating that “the main differences between sections *Prosopis*, *Algarobia* and *Strombocarpa* Benth. are vegetative spine characters and are, therefore, only of subgeneric rank” (Burkart 1976: 227), even though he acknowledged that the variation in armature probably had phylogenetic significance (see below).

**Figure 1.**
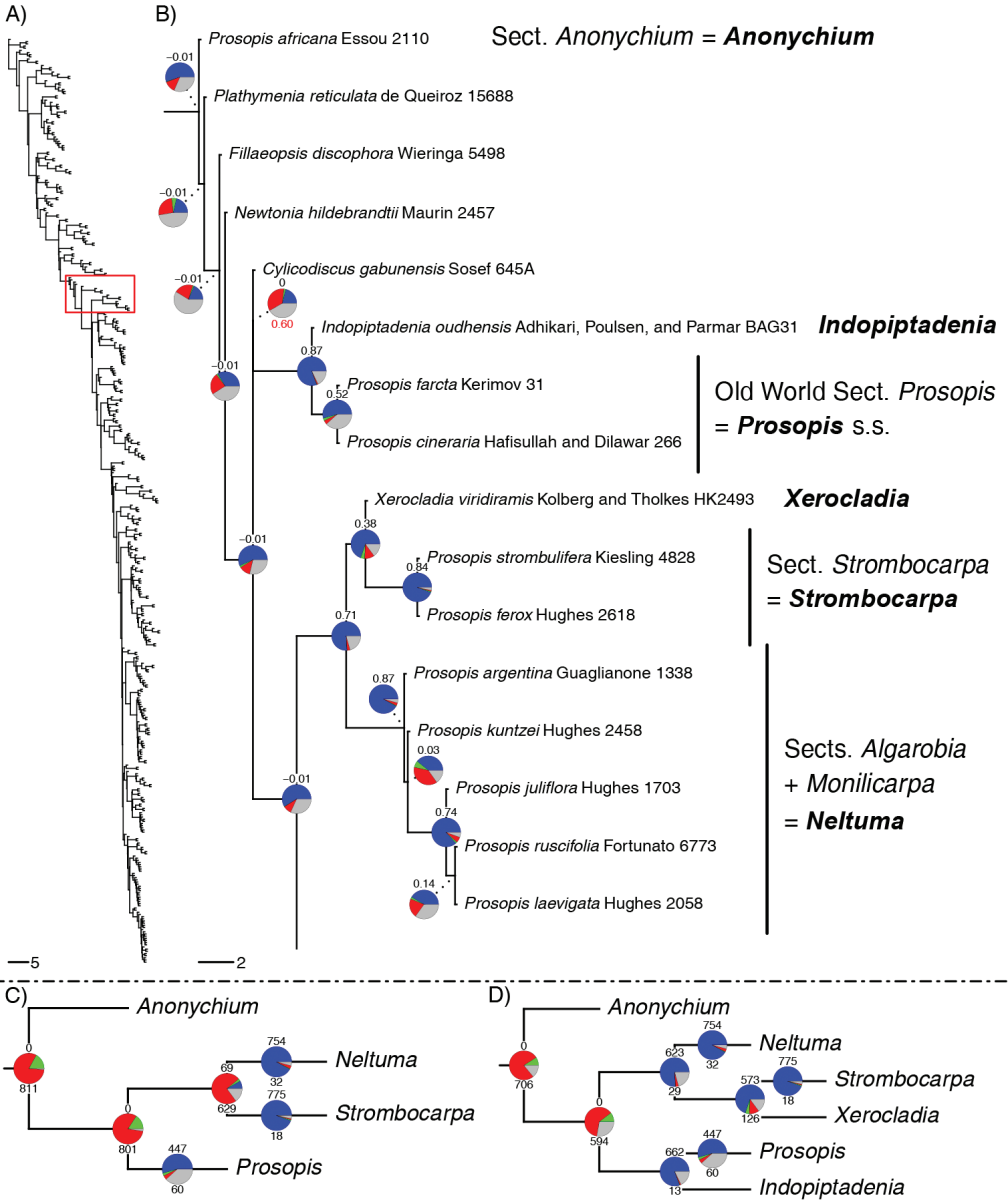
**A** Phylogeny of the Caesalpinioideae showing the placement of the *Prosopis* grade (boxed in red) within the subfamily, based on analyses of DNA sequences of 997 nuclear genes (Ringelberg et al. 2022) **B** the part of the phylogeny that includes all elements of *Prosopis* s.l. Genera recognised in the new generic system presented here are in **bold**. Pie charts show the fraction of gene trees supporting that bipartition in blue, the fraction of gene trees supporting the most likely alternative configuration in green, the fraction of gene trees supporting additional conflicting configurations in red and the fraction of uninformative gene trees in grey. Numbers above pie charts are Extended Quadripartition Internode Certainty (Zhou et al. 2020) scores. Branch lengths are expressed in coalescent units and terminal branches were assigned an arbitrary uniform length for visual clarity, see Ringelberg et al. (2022); the root is not drawn to scale **C, D** the two most likely alternative tree topologies which would allow for a monophyletic *Prosopis* s.l., either without (**C**) or with (**D**) *Xerocladia* and *Indopiptadenia*. In **C** and **D** numbers above pie charts = number of gene trees supporting the species tree, numbers below pie charts = number of gene trees conflicting with the species tree **C** lack of gene tree support (just 69 gene trees) for the alternative species tree topology where sections *Algarobia* + *Monilicarpa* (≡ *Neltuma*) are sister to section Strombocarpa (≡ *Strombocarpa*) vs. 573 genes supporting a sister group relationship between *Strombocarpa* and *Xerocladia* (as shown in **D**) **D** lack of gene trees (zero gene trees) supporting a monophyletic *Prosopis* s.l.

This long-held generic concept of *Prosopis* established by Bentham (1842, 1875) and followed by Burkart in his 1976 monograph, is no longer sustainable, because molecular phylogenies have demonstrated, beyond doubt, that *Prosopis* is polyphyletic. This non-monophyly was first revealed by Catalano et al. (2008) and confirmed by LPWG (2017) who showed that *P.africana* (Guill. & Perr.) Taub. forms an isolated monospecific lineage quite separate from the rest of *Prosopis* and that the monospecific Namibian/S. African endemic genus *Xerocladia* Harv. was potentially nested within *Prosopis*, but these analyses lacked robust support and sampling of critical taxa. Recent phylogenomic analyses of a much larger DNA sequence dataset, based on 997 nuclear genes (Koenen et al. 2020) that now includes all but five of the 152 genera of Caesalpinioideae (Ringelberg et al. 2022), have confirmed this non-monophyly showing robust support for three separate lineages (Fig. 1): (i) a lineage comprising *P.africana*, which is placed in a grade made up of the genera *Plathymenia* Benth., *Fillaeopsis* Harms and *Newtonia* Baill., as found by Catalano et al. (2008); (ii) a lineage comprising the remaining Old World species of *Prosopis* which is robustly supported as sister to the monospecific genus *Indopiptadenia* Brenan from the Himalayan foothills of the Terai border region of Nepal and India (Bajpai et al. 2014); (iii) a lineage comprising the New World species of *Prosopis* plus the Namibian/South African endemic genus *Xerocladia*, which is nested within this clade, again confirming the preliminary results of Catalano et al. (2008). The DNA sequence dataset of Ringelberg et al. (2022), based as it is on a large number of nuclear genes, can also be used to quantify how many genes support a particular species tree topology and, thereby, how robust the phylogeny is (Fig. 1B) and also how many genes support alternative species tree topologies. These analyses show that just 69 gene trees support a sister group relationship between sections *Strombocarpa* (= *Strombocarpa*) and *Algarobia* + *Monilicarpa* Ruiz Leal & Burkart (= *Neltuma* Raf.), while 629 of the gene trees conflict with that topology (Fig. 1C) and none of the gene trees supports a monophyletic *Prosopis* s.l. (Fig. 1D), confirming that there is an overwhelming number of gene trees supporting the species tree topology in Fig. 1B. It is thus now clear that maintaining *Prosopis* in its current circumscription is untenable.

**Figure 2.**
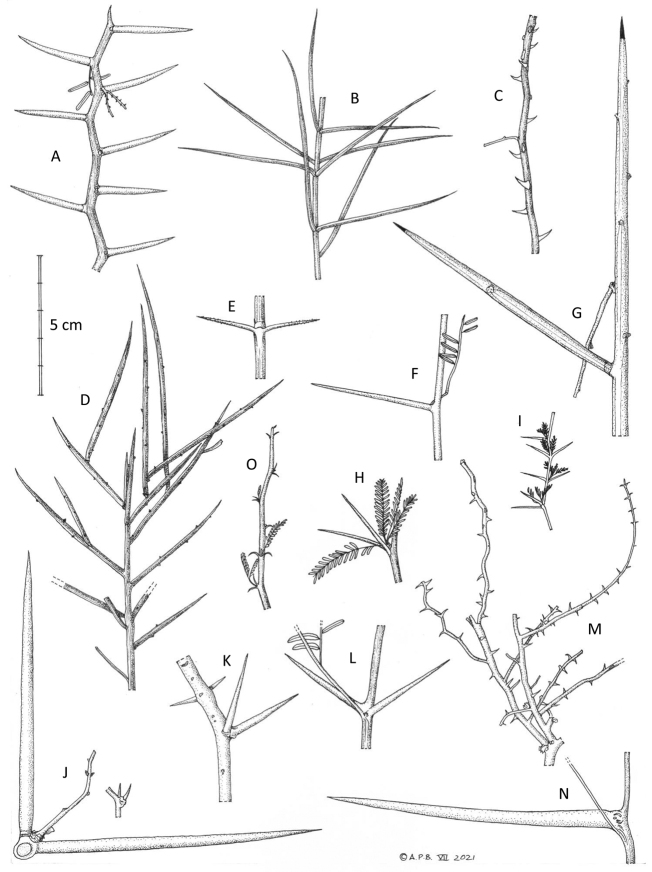
Variation in armature of *Prosopis*, *Strombocarpa*, *Neltuma* and *Xerocladia***A***Neltumadenudans* (nodal spines on a zig-zag stem) **B***N.humilis* (paired striate spine-tipped branches) **C***Prosopiscineraria* (scattered internodal prickles) **D***Neltumasericantha* (spine-tipped stems) **E***Strombocarpaburkartii* (stipular spines) **F***Neltumaargentina* (single nodal axillary spine) **G***N.kuntzei* (spinescent shoots) **H***Strombocarpaferox* (stipular spines) **I***S.strombulifera* (stipular spines) **J***Neltumaelata* (variation in paired nodal spines on one specimen) **K***N.alba* (paired nodal spines) **L***N.velutina* (paired nodal spines) **M***Prosopisfarcta* (scattered internodal prickles) **N***Neltumaruscifolia* (single nodal axillary spine) **O***Xerocladiaviridiramis* (recurved, deflexed stipular spines) (5 cm scale bar). All specimens at K **A** drawn from *Seijo* 1489 **B***Tweedie* s.n. **C***Willcox* 299 **D** MERL 8792 **E***Acosta & Rosas* 748 **F***Guaglianone et al.* 1762 **G***Nee & Coimbra* 35556 **H***Atahuachi* et al. MA1147 **I***Hunziker* 2036 **J***Legname & Cuezzo* 10396 (large and small spines from same specimen) **K***Hughes & Forrest* 2312 **L***Harding & Balsinhas* 140 **M***Guest et al.* 17463 **N***Wood & Mamani* 14063 **O***Kolberg & Tholkes* HK2493. Drawn by Andrew Brown, July 2021.

**Figure 3.**
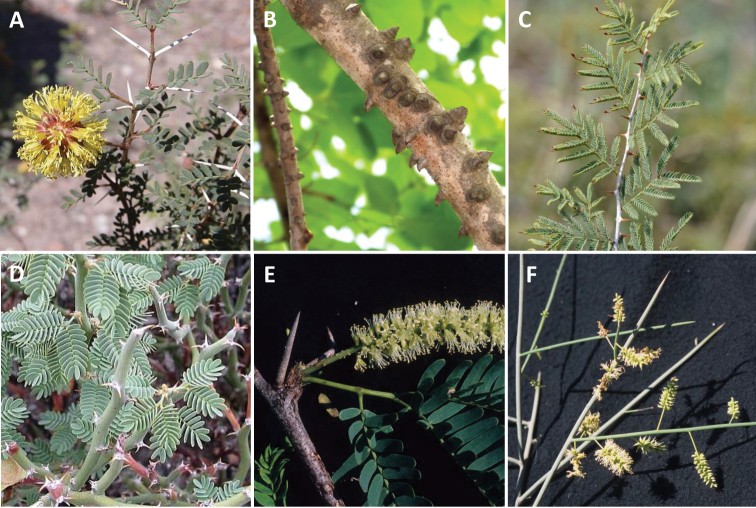
Variation in armature across *Prosopis* s.l. and allies **A** stipular spines of *Strombocarpastrombulifera***B** internodal prickles on shoots and branches of *Indopiptadeniaoudhensis* which it shares with its sister group, *Prosopis* s.s. illustrated in **C**; **C** internodal prickles of *Prosopisfarcta***D** stipular spines of *Xerocladiaviridiramis* which it shares with its sister group, the genus *Strombocarpa* illustrated in **A**; **E** axillary nodal spines of *Neltumajuliflora***F** spinescent straight cylindrical shoots of the subaphyllous *Neltumakuntzei*. Photos courtesy of Guillermo Debandi (**A**) (see https//www.inaturalist.org/taxa/78750-Prosopis-strombulifera/browse_photos), Dr. Omesh Bajpai and Dr. Lal Babu Chaudhary (**B**), Zeynel Cebeci (**C**) (see https//commons.wikimedia.org/wiki/FileProsopis_farcta_-_Syrian_mesquite_01), N. Dreber (**D**) (see http//www.southernafricanplants.com/), Colin Hughes (**E, F**).

**Figure 4.**
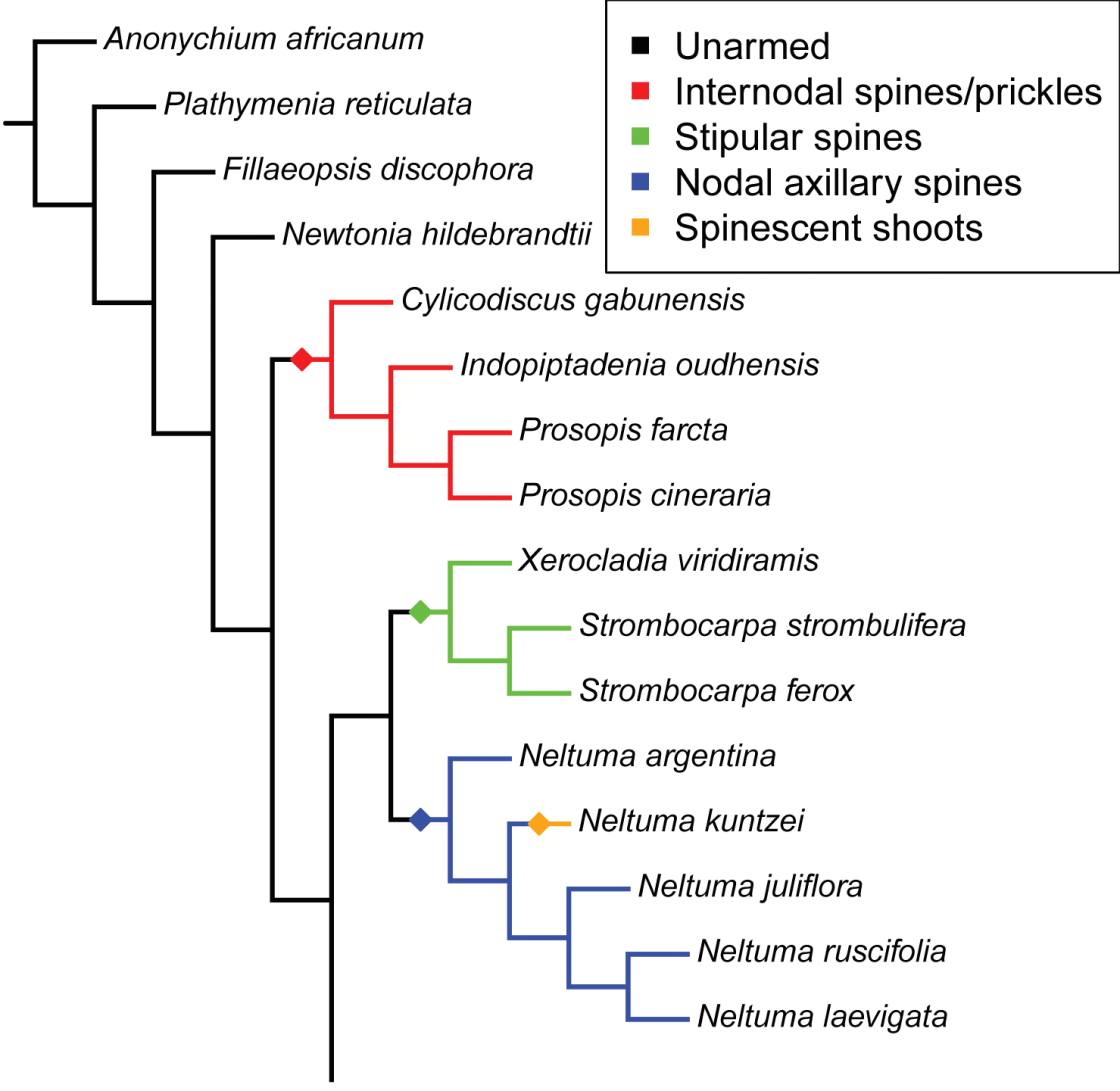
Independent evolutionary origins of stipular spines, axillary nodal spines and internodal spines across the segregate genera of the *Prosopis* s.l. grade. Diamonds indicate putative origins halfway along the branch subtending the clade with the character of interest. Note that, in the case of *Neltumakuntzei*, a loss of axillary nodal spines, which are absent in that species, apparently coincides with an evolutionary gain of spinescent shoots (see also Fig. 3F) and with a shift to a largely aphyllous condition on the mature shoots. The reconstruction of armature characters shown here encompasses results of four independent optimisations of four types of armature, performed using the make.simmap option of R (R Core Team 2021) package phytools (Revell 2012), each with 500 simulations using the ARD model. Optimisations were performed on an ASTRAL phylogeny of the entire Caesalpinioideae, based on 821 single-copy genes (Ringelberg et al. 2022), but are here shown only for the *Prosopis* s.l. grade with standardised branch lengths.

What is immediately striking from Fig. 1 and the earlier phylogeny of Catalano et al. (2008) with its denser sampling of species across New World *Prosopis*, is that these three separate lineages of *Prosopis* species correspond to and are congruent with Burkart’s sections (apart from the inclusion of *Xerocladia*) and with the variation in armature upon which Burkart’s sections were based (Figs 2–4): Section Anonychium Benth. = *P.africana*, is unarmed in common with the rest of the grade of lineages (*Plathymenia*, *Fillaeopsis* and *Newtonia*) that subtend the large core mimosoid clade of Koenen et al. (2020) (Fig. 1; Ringelberg et al. 2022); Section Prosopis = the rest of Old World *Prosopis*, comprising *P.cineraria* (L.) Druce, *P.farcta* (Banks & Sol.) J.F. Macbr. and *P.koelziana* Burkart (from Iran), all have straight internodal prickles (Figs 2C, M and 3C), which are also found in the sister genus of this clade, *Indopiptadenia*, including in the form of large, conical, hard, sharp-pointed spines on older stems and trunk (Fig. 3B; see also Bajpai et al. 2014: figs 2B–H and 11A); species of Section Strombocarpa plus the genus *Xerocladia* have stipular spines (Figs 2E, H, I, O and 3A, D); and species of sections *Monilicarpa* + *Algarobia* variously have spinescent shoots or uninodal axillary solitary or geminate spines (Figs 2A, B, D, F, G, J–L, N and 3E, F and 4), but never the internodal prickles of section Prosopis, nor the stipular spines of section Strombocarpa (see also Benson 1941). These three types of armature are non-homologous, even though they have evolved to meet similar plant defence functions and can look superficially similar. To explore the evolution of armature across the *Prosopis* s.l. grade, we scored these different types of armature across genera of subfamily Caesalpinioideae and optimised these on to the Ringelberg et al. (2022) phylogeny. This reconstruction shows independent derivations of stipular spines, internodal prickles and axillary nodal spines (Fig. 4), each providing diagnostic synapomorphies for clades in the context of *Prosopis* s.l. (Fig. 4). Ironically, in his justification of the unity of *Prosopis*, Burkart (1976) pointed to *Acacia* Mill. s.l. as another group that also showed considerable diversity in types of armature and other vegetative traits, but which was considered (at that time) to comprise a single genus. Given that *Acacia* s.l. was later demonstrated to be polyphyletic (reviewed by Maslin et al. 2003) and has now been dismantled into seven segregate genera, several of which are distinguished primarily by differences in armature (e.g. the stipular spines that distinguish *Vachellia* Wight & Arn. from the cauline nodal and internodal prickles of *Senegalia* Raf.), Burkart’s suggestion that a wide concept of *Acacia* chimed with his wide concept of *Prosopis* can now be seen with hindsight to have been misplaced.

**Figure 5.**
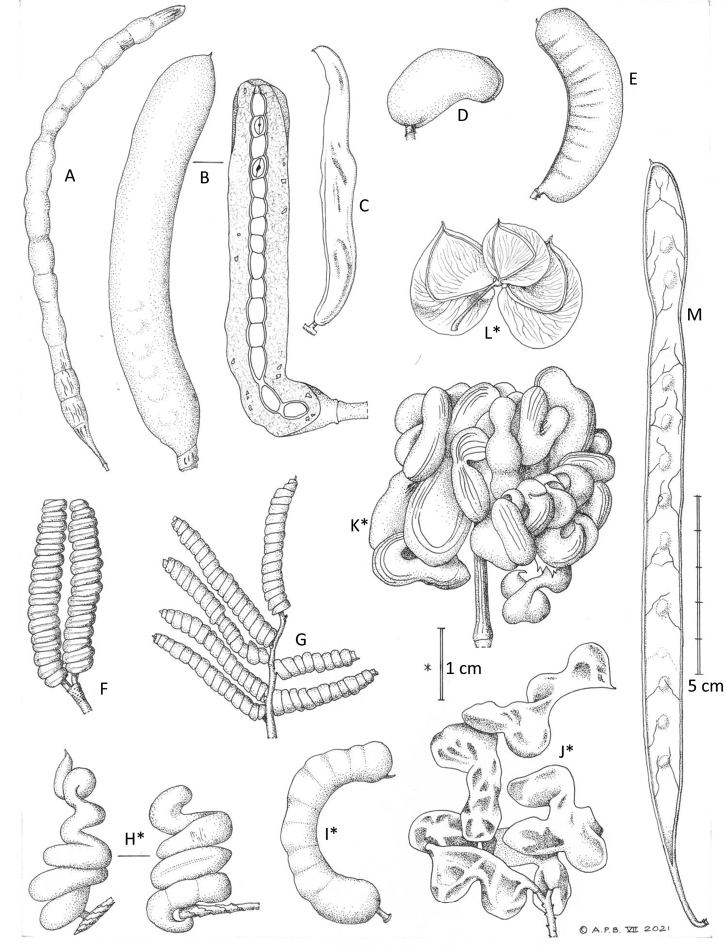
Fruits of *Prosopis*, *Strombocarpa*, *Xerocladia* and *Indopiptadenia***A***Prosopiscineraria***B***Anonychiumafricanum***C***Strombocarpapalmeri***D***Prosopisfarcta***E***Strombocarpaferox***F***S.strombulifera***G***S.pubescens***H***S.abbreviata* (2 examples) **I***S.tamarugo***J***S.torquata***K***S.burkartii***L***Xerocladiaviridiramis***M***Indopiptadeniaoudhensis***A-G, M** (5 cm scale bar) **H-L** (1 cm scale bar with asterisk). All specimens at K **A** drawn from *Gazanfar* SG4332 **B***Dembele & Sanogo* ML-146 and longitudinal section of fruit from *Barter* 1193 **C***Hughes et al.* 1552 **D***van der Maesen* 1627 **E***Atahuachi et al*. MA1147 **F***Hunziker* 2036 **G***Acocks* 1788 **H***Tweedie* s.n. (from 2 type specimens) **I***Aronson* 7742 **J***Vuilleumier* 1019 **K***Acosta & Rosas* 748 **L***Kolberg & Tholkes* HK2493 **M***Bajpai & Babu* 264498. Drawn by Andrew Brown, July 2021.

**Figure 6.**
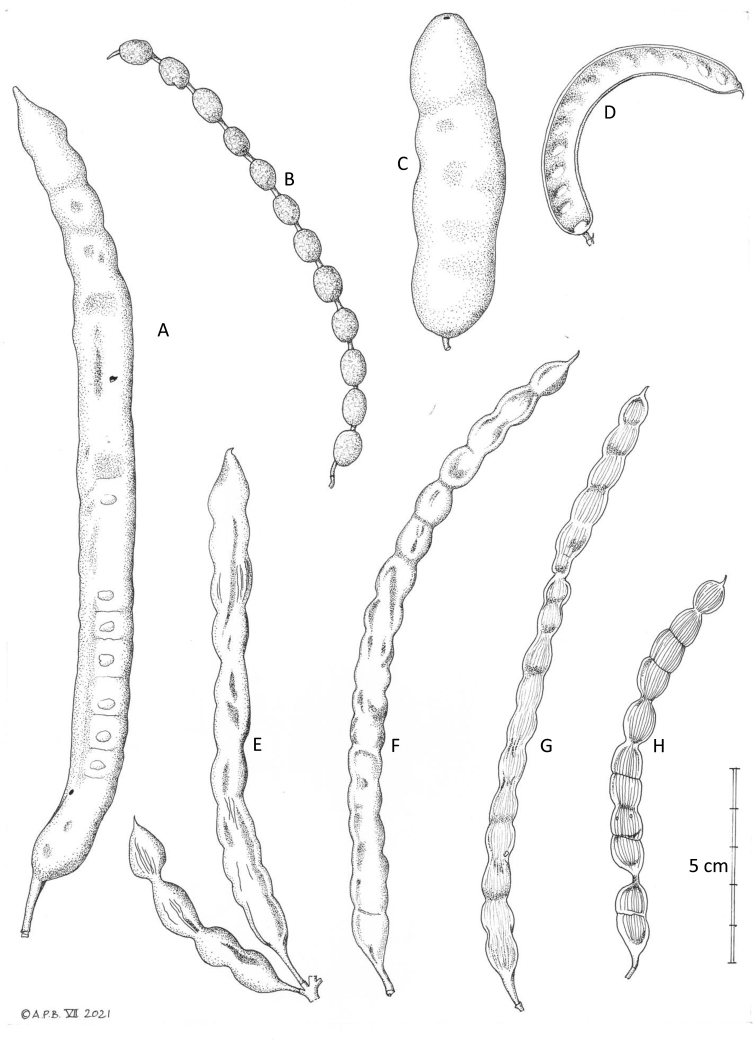
Fruits of *Neltuma***A***Neltumaalba***B***N.argentina***C***N.kuntzei***D***N.denudans***E***N.laevigata***F***N.nigra***G***N.articulata***H***N.ruscifolia*.(5 cm scale bar). All specimens at K **A** drawn from *Hughes & Forrest* 2312 **B***Guaglianone et al.* 1762 **C***Nee & Coimbra* 35556 **D***Seijo* 1489 **E***Manríquez & Tenorio* 6563 **F***Arenas* 3123 **G***Hughes et al.* 1559 **H***Wood & Mamani* 14063. Drawn by Andrew Brown, July 2021.

**Figure 7.**
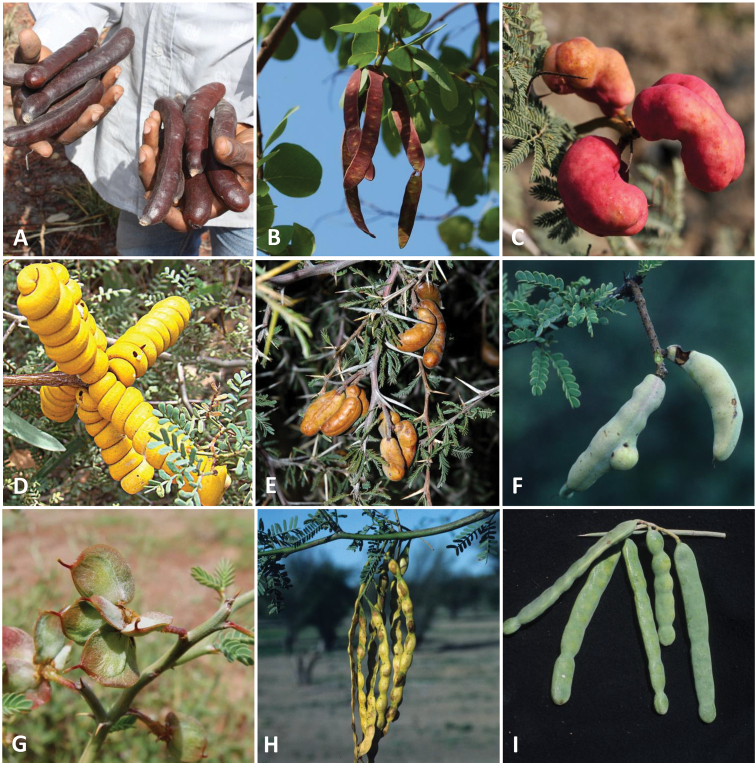
Variation in fruits across *Prosopis* s.l. and allies **A** indehiscent pods of *Anonychiumafricanum* with thick pulpy mesocarp collected as fodder for livestock **B** plano-compressed pods of *Indopiptadeniaoudhensis* lacking a thickened mesocarp and dehiscent along both sutures **C** indehiscent fruits of *Prosopisfarcta* with a thick pulpy mesocarp **D** tightly coiled indehiscent screwbean fruits of *Strombocarpastrombulifera***E** indehiscent pods of *Strombocarpaferox* with a thick pulpy mesocarp **F** indehiscent fruits of *Strombocarpapalmeri***G** small reniform to flabellate, flattened, indehiscent, 1 (–2)-seeded, winged fruits of *Xerocladiaviridiramis* which are unique within mimosoid legumes **H** indehiscent fruits of *Neltumaarticulata* with a thick mesocarp and a hard bony segmented endocarp which remains closed **I**. Unripe indehiscent pods of *Neltumakuntzei* with a thick pulpy mesocarp, these turning dark blackish-brown when ripe, reminiscent in colour to fruits of *Anonychium*. Photos courtesy of Marco Schmidt (**A**) (see Dressler et al. 2014), Dr. Omesh Bajpai and Dr. Lal Babu Chaudhary (**B**), Zeynel Cebeci (**C**) (https//en.wikipedia.org/wiki/Prosopis_farcta), Dick Culbert (**D**) (see https//eol.org/pages/640506, Colin Hughes (**E, F, H, I**), and Herta Kolberg (**G**) (see Plants of Namibia https//herbaria.plants.ox.ac.uk/bol/namibia).

The apparent phylogenetic significance of types of armature to distinguish important clades and genera across Caesalpinioideae, contrasts with the striking evolutionary lability of fruit types, as seen across *Prosopis* s.l. and allies (Figs 5–7). This is exemplified by the contrast between the cylindrical or sub-cylindrical thickened indehiscent fruits of *Prosopis* s.l. (albeit varying considerably in the degree to which they are curved or coiled (see below)) and the very different plano-compressed fruits of *Indopiptadenia* (Figs 5M and 7B; see also Bajpai et al. 2014: Fig. 7), which is sister to section Prosopis and which lacks a thickened mesocarp and is dehiscent along one or both sutures. Similarly, *Xerocladia*, which is sister to section Strombocarpa (Fig. 1), has equally distinctive small reniform to flabellate, flattened, indehiscent, 1 (–2)-seeded, winged fruits, which are unique amongst mimosoid legumes as a whole (Figs 5L and 7G) and also lack the often-thick mesocarp of *Prosopis* s.l. fruits (Gunn 1984). Thus, it is now clear that the thickened, sub-cylindrical fruits of *P.africana* (section Anonychium), which are superficially very similar (both are thick, woody, indehiscent and black when mature) to those of distantly related *P.kuntzei* Harms (section Algarobia) (Figs 5B, 6C, 7A, I), represent homoplasious evolutionary origins of similar endozoochorous seed dispersal syndromes, based on animal ingestion of highly palatable fruits and defecation of the seeds (Tybirk 1991; Weber et al. 2008) and, hence, are misleading as the basis for generic delimitation. In the light of phylogenetic data, it is now clear that Burkart’s (1976) reliance on fruit morphology to unite his broad concept of *Prosopis* and demotion of armature as only useful at sub-generic rank and not for delimiting genera were misplaced.

It is notable that pollen exine structure also supports these groups. Pollen of the Old World species of section Prosopis is similar to that of its sister genus *Indopiptadenia*, showing a relatively thin (0.7–0.9 µm) tectum with irregularly areolate-verrucose raised sculpturing, whereas the New World species of *Prosopis*, and *Xerocladia* have a smooth (perforated) and even thinner (< 0.7 µm) tectum (Hernández and Guinet 1990: Fig. 5).

The type species of *Prosopis*, *P.spicigera* L. (a synonym of *P.cineraria* (L.) Druce), is from the Old World in section Prosopis of Burkart (1976), a clade that comprises just three of the 56 species currently recognised in the genus as a whole, implying that the remaining 53 species will require a name change to deal with the non-monophyly of *Prosopis* s.l. Segregation of the isolated monospecific lineage *P.africana* as a separate genus presents a straightforward and uncontroversial adjustment, here implemented by re-instatement of the genus *Anonychium* (Benth.) Schweinf. (see below). Generic re-delimitation of the New World species is less straightforward and is complicated by placement of the morphologically distinctive Namibian/Namaqualand monospecific genus *Xerocladia* nested within the New World Prosopis clade as sister to section Strombocarpa (Fig. 1). Despite its similar shrubby, multi-stemmed, branchy habit, green shoots, stipular spines (Fig. 3D) (shared with section Strombocarpa) and occurrence in arid succulent-rich vegetation, all of which are shared with New World *Prosopis*, the genus *Xerocladia* has been maintained as distinct from *Prosopis*, because it has highly distinctive reniform to flabellate, indehiscent, 1(–2)-seeded, winged fruits (Figs 5L and 7G), lacking a thickened mesocarp, which are very different from those of *Prosopis* s.l. and, indeed, from all other mimosoid legumes. Given this distinctive morphology, we retain *Xerocladia* as a separate genus. We also note that the material referred to under the name *Xerocladiapampeana* Speg. from Argentina, shows clear affinities to the genus *Prosopidastrum* Burkart, as suggested by Palacios and Hoc (2005). Even though Palacios and Hoc (2005) left *X.pampeana* as an excluded name in their treatment of Argentinian *Prosopidastrum*, examination of the material cited by them suggests that the fruits are not monospermous, but simply broken fragments of lomentiform fruits of *Prosopidastrum*.

Retention of the monospecific African *Xerocladia* at generic rank implies that the two subclades of New World *Prosopis* species, corresponding to Sections *Strombocarpa* and *Monilicarpa* + *Algarobia* of Burkart (1976) (Fig. 1), also need to be recognised as separate genera. Both of these groups have been previously ranked as genera. Bentham (1839), prior to uniting the various elements of *Prosopis* s.l. in a single genus in his 1875 treatment of Mimoseae, recognised section Algarobia at generic rank as the genus *Algarobia* Benth. (even though the name *Algarobia* is preceded by *Neltuma* Raf. published one year earlier in 1838). Similarly, section Strombocarpa was also afforded generic status as the genus *Strombocarpa* Englm. & A. Gray in 1845, a generic delimitation followed by Britton & Rose (1928) in their treatment for the North America Flora. The alternative to recognising these two New World clades as separate genera would be to transfer all New World species of *Prosopis* plus the African *Xerocladia* to the genus *Neltuma*. While it could be argued that this alternative would make generic-level identification in the New World easier, it would entail lumping *Xerocladia* with its highly unusual fruits which are unique within mimosoids and would detract from the overall ability to diagnose genera across mimosoids. We believe that upranking Burkart’s sections *Strombocarpa* and *Algarobia* + *Monilicarpa* as the genera *Strombocarpa* and *Neltuma*, respectively, distinguished by the differences in armature that provided the basis for Burkart’s sections, while retaining the African *Xerocladia* as a separate genus (Fig. 1), provides the best solution to render all genera monophyletic and ensure maximal ability to diagnose genera across mimosoids as a whole.

Finally, for completeness, we note that the genus *Sopropis* Britton & Rose, erected by Britton & Rose (1928) to accommodate the somewhat unusual species *Sopropispalmeri* (S. Watson) Britton & Rose (= *Prosopispalmeri* S. Watson) has the stipular spines of section Strombocarpa, but a straight (or only weakly falcate) fruit more typical of section Algarobia (Figs 5C and 7F), as noted by Benson (1941). In the phylogeny of Catalano et al. (2008), *P.palmeri* is placed in the clade corresponding to section Strombocarpa with robust support, vindicating the congruence of armature types across the phylogeny and we here treat *Sopropis* as a synonym of *Strombocarpa*. This is very much in line with Burkart’s (1976) view that too much weight had been given by Britton and Rose (1928) to the curvature and coiling of the *Prosopis* fruit in the recognition of three distinct genera in their Flora of North America treatment. Indeed, it is clear that curvature of the pod across New World *Prosopis* s.l. shows a continuum from the tightly spirally coiled ‘screwbean’ pods of, for example, *P.strombulifera* (Lam.) Benth. and *P.pubescens* Benth. (Figs 5F, G and 7D), to fruits with fewer, larger and more open coils, annular fruits and those that are only weakly curved or completely straight, variation that is discordant with sectional boundaries (Figs 5B, C, E–K, 6 and 7D–F, H–I) and with the phylogeny (Fig. 1).

**Figure 8.**
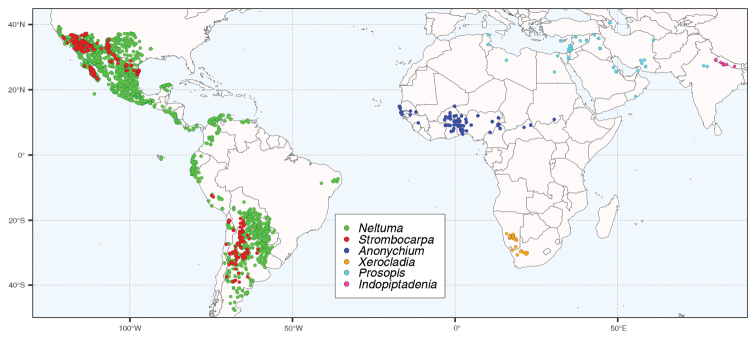
The distributions of *Indopiptadenia*, *Prosopis* s.s., *Anonychium*, *Xerocladia*, *Neltuma* and *Strombocarpa*, based on 6,469 quality-controlled species occurrences from GBIF (www.gbif.org), DryFlor (www.dryflor.info), SEINet (www.swbiodiversity.org/seinet) and several other data sources (Ringelberg et al., in prep.). Map created using R packages ggplot2 (Wickham 2016), sf (Pebesma 2018) and rnaturalearth (South 2017). The eight occurrence records, mapped in Bahia Brazil, are of *Neltumaruscifolia* which is considered potentially native to that region (Burkart 1976 Oliveira & Queiroz 2020), while records of *N.juliflora* from Bahia, which is introduced and naturalised in that region, have been eliminated.

Taxonomic name changes are often unwelcome for many users, at least in the short term, especially for plant groups that are important ecologically and in terms of human uses. This is very much the case for *Prosopis* s.l. and especially so in the warm desert and dryland scrub ecosystems of the New World, where “few plant genera have received as much attention as *Prosopis*” (Simpson 1977: ix). Species of *Prosopis* are ecologically abundant in many parts of its New World range, dominating vast tracts of the Chaco in South America and the matorrales of the southern U.S.A. and parts of Mexico (Fig. 8) (Benson 1941). Trees of *Prosopis* s.l. also occupy a central place in silvo-pastoral systems more widely across the arid and semi-arid tropics from Rajasthan in NW India, through the Arabian Peninsula, across Sahelian Africa and throughout the arid zones of the Neotropics (Leakey and Last 1980; Fagg and Stewart 1994; Pasiecznik et al. 2001; Weber et al. 2008), because of their dependable provision of abundant protein- and sugar-rich, non-toxic, highly palatable and nutritious fruits during the dry season that are eagerly consumed by diverse livestock (cattle, sheep, goats, camelids). Furthermore, *Prosopis* s.l. fruits, including the *mezquites* in North America (Felger 1977) and the *algorrobos* in South America (D’Antoni and Solbrig 1977), constituted one of the most important wild food sources for pre-hispanic cultures, with *P.velutina* Wooton, the velvet mesquite referred to as the ‘tree of life’ (Bell and Castetter 1937) and these uses potentially prompting long distance translocation of species by humans and their livestock within the Americas in pre-Colombian times (McRostie et al. 2017). In addition to livestock fodder and human food, the wood of *Prosopis* is dense and durable and widely used for firewood, charcoal and parquet flooring and the flowers provide high quality, reliable and abundant forage for honey bees. Moreover, such is the ability of some *Prosopis* species to disperse seeds, colonise and quickly form dense spiny impenetrable thickets, that some species of *Prosopis* are amongst the world’s worst invasive weeds, both within and well beyond their native ranges. For example, several New World section Algarobia species are naturalised and invasive across many parts of Africa, the Middle East, the Indian subcontinent and Australia (e.g. Pasiecznik et al. 2001; Van Klinken and Campbell 2001; Ayanu et al. 2015) and have been recorded from 103 countries and considered to be invasives in 49 of those (Shackleton et al. 2014). Within their native distributions, *P.ruscifolia* Griseb. is a serious pest in the western Gran Chaco, referred to as a ‘*plaga nacional*’ and *P.glandulosa* Torr. has prompted the so-called ‘*mesquite problem*’ in Texas in the southern U.S.A. where that species is considered a serious rangeland weed (Fisher et al. 1959).

The impacts of name changes on a group of plants of such diverse importance cannot be denied and, inevitably, we anticipate resistance, in the short term, to the nomenclatural changes we propose here. Notwithstanding, we also expect that, ultimately, there will be benefits from aligning genera with monophyletic groups that more accurately reflect their evolutionary placements and provide a deeper biological understanding of these globally-important plants. In that light, it is notable that all the serious invasive and rangeland pest species fall into *Neltuma* (= section Algarobia), suggesting that a propensity for invasiveness is more problematic for species in that clade. Similarly, of 29 species of bruchid beetles known to predate seeds of New World *Prosopis*, only two span *Neltuma* (sections *Algarobia* + *Monilicarpa*) and *Strombocarpa*, such that each of the two New World clades has largely its own exclusive bruchid fauna, including, for example, the bruchid genus *Algarobius* Bridwell which is largely restricted to species of section Algarobia (Kingsolver et al. 1977). More generally, biocontrol programmes to mitigate invasions of New World species of *Neltuma* in Africa have focused on insects, such as the bruchid seed predator *Algorobiusprosopis* (J.L. Leconte), that do not attack native African members of *Prosopis* s.l. including species of *Prosopis* s.s., *Anonychium* and *Xerocladia*, suggesting that many insects effectively distinguish amongst the genera proposed here (Kleinjan et al. 2021).

It is also notable that, while intra-sectional interspecific hybridisation has been reported to occur in both section Strombocarpa (e.g., the hybrid origin of *Prosopisburkartii* Muñoz, Contreras et al. 2020) and amongst a subset of species in the ‘mesquite clade’ of *Neltuma* (= sections *Algarobia* + *Monilicarpa*) (Hunziker et al. 1986; Castillo et al. 2021), there are no examples of inter-sectional hybrids between species belonging to *Neltuma* and *Strombocarpa* (Solbrig et al. 1977; Hunziker et al. 1986), despite their sympatry across many areas (Fig. 8). This lack of inter-sectional crossing prompted Hunziker et al. (1986) to suggest upranking sections *Algarobia* (= *Neltuma*) and *Strombocarpa* “at least to the level of subgenera”, as also suggested by Saidman et al. (1996), based on genetic differences. Similarly, phylogenetic analysis of morphology and biochemical traits showed strong support for recognising *Strombocarpa* as a distinct clade (Burghardt and Espert 2007). These trait differences, alongside other ecological differences, are symptomatic of the deep (phylo)genetic split between these two clades which are estimated to have diverged 25 Myr (Ringelberg et al., in prep). All these differences in biology are of potential significance for genetic improvement, range management and biocontrol programmes (see Kleinjan et al. 2021), adding further justification to recognise Burkart’s sections at generic rank.

## ﻿Biogeography

One of the uniting features of *Prosopis* s.l. is the distribution of its various lineages, first and foremost, in seasonally dry and arid tropical and subtropical climates across the New and Old Worlds (Fig. 8), a distribution that spans, in large part, the transcontinental grass-poor succulent-rich, fire-free succulent biome sensu Schrire et al. (2005) and Ringelberg et al. (2020). However, in that sense, *Anonychium* (*P.africana*) is an outlier, just as it is phylogenetically, because it grows in savannahs across Sahelian Africa. The *Prosopis* s.s. + *Indopiptadenia* clade spans an interesting dry/monsoonal west-central Asian distribution which is unique amongst mimosoids. At first sight, the sister group relationship between *Strombocarpa* and *Xerocladia* spanning the Atlantic seems a surprising disjunction, but several other Caesalpinioid legumes show similar disjunct amphi-Atlantic distributions with most of their diversity in the Neotropics and outlying endemic species in Namibia and adjacent regions of southern Africa. These include the genera *Haematoxylum* L., *Parkinsonia* L. and *Pomaria* Cav., with *Haematoxylumdinteri* Harms, *Parkinsoniaafricana* Sond. and three species of *Pomaria* in Namibia and S. Africa. Two things are notable about these transatlantic disjunctions. First, they often show bicentric amphitropical ranges in the New World and disjunctions in SW Africa (the *Haematoxylum* + *Lophocarpinia* Burkart clade; *Pomaria* (Simpson et al. 2006); *Strombocarpa* - *Xerocladia*). Second, they share similar seasonally dry tropical, grass-poor, succulent-rich, fire-free ecologies across the transcontinental Succulent Biome (Schrire et al. 2005; Gagnon et al. 2019; Ringelberg et al. 2020).

### ﻿Key for the identification of the segregate genera of *Prosopis* and close allies (see Figs 2 and 3 for illustrations of armature characters used in the key)

**Table:** 

1	Plants unarmed	***Anonychium***
–	Plants usually armed with stipular spines, axillary solitary or paired uninodal cauline spines, spinescent shoots or internodal prickles	**2**
2	Plants armed with internodal prickles on shoots and/or stems, petals glabrous	**3**
–	Plants armed with stipular spines, axillary solitary or paired uninodal spines or spinescent shoots, petals villous or pilose	**4**
3	Fruits indehiscent, cylindrical or subterete, with a pulpy or fibrous mesocarp, largest leaflets < 1.5 × 1 cm, mature stems with scattered prickles	***Prosopis***
–	Fruits dehiscent, plano-compressed, coriaceous, lacking a thick mesocarp, larger leaflets > 3 × 3 cm, mature stems with spine-tipped woody protuberances	***Indopiptadenia***
4	Fruits reniform to flabellate, indehiscent, 1–2-seeded and winged	***Xerocladia***
–	Fruits linear or oblong, always > 2-seeded	**5**
5	Plants armed with stipular spines	***Strombocarpa***
–	Plants armed with axillary, uninodal, solitary or paired spines or spinescent shoots	***Neltuma***

## ﻿Taxonomy

We present a taxonomic synopsis of the four segregate genera, *Anonychium*, *Prosopis*, *Strombocarpa* and *Neltuma*, including 57 new nomenclatural combinations and associated synonymy. Type details are cited for accepted names, but not for heterotypic synonyms.

### ﻿ Anonychium

Taxon classificationPlantaeFabalesLeguminosae

(Benth.) Schweinf., Reliq. Kotschy.: 7. 1868.

FFEAC5AA-F99D-5078-BFD3-C78FD31A69BF

Prosopis section Anonychium , Benth. Hook. J. Bot. 4: 347. 1842.

#### Type.

*Prosopisoblonga* Benth. Benth., J. Bot. (Hooker) 4: 348. 1842, a synonym of *Anonychiumafricanum*.

#### Description.

Unarmed trees 4–20 m high, branches lacking axillary brachyblasts. Stipules inconspicuous, long-lanceolate, pubescent, caducous as young leaves develop, absent from most herbarium sheets. Leaves somewhat pendulous, 1–4 pairs of pinnae, the petiole 3–5 cm long, the rachis 5–9 cm long, the pinnular rachises 6–15 cm long, with 4–13 pairs of opposite leaflets, these 1.3–3.5 × 0.4–1.5 cm, glabrous or finely pubescent, mid-vein subcentric. Inflorescences spicate, 5–9 cm long, axillary, solitary or in pairs, densely flowered; pedicels 0.5 mm. Flowers small, yellowish or greenish-white, sweetly scented; calyx ca. 1 mm long; corolla ca. 3.5 mm long, the petals linear, free, glabrous on both sides; anthers apically broadened with an unusual anther gland borne ventrally between the thecae and forming a triangular hood-shaped protrusion made up of papillate cells; pollen with costae on the pores and a smooth (perforated) tectum; ovary and style pilose or villous. Fruits indehiscent, straight or sub-falcate, dark reddish-brown to blackish, shiny, subterete, 10–20 × 1.5–3.3 cm, exocarp hard, 1–2 mm thick, mesocarp spongy, thick, dry, endocarp segments thin, longitudinal, in one row (Figs 5B and 7A). Seeds many, dark, shiny, ovate compressed, 8–10 × 4–9 mm, rattling within the pod when ripe.

#### Geographic distribution.

Monospecific. Widespread across Sahelian Africa, from Senegal in the west to Sudan and Ethiopia in the east (Fig. 8).

#### Habitat and uses.

*Anonychiumafricanum* is native across the whole Sahelian savannah belt. Trees are maintained and managed by farming and pastoralist communities in traditional silvo-pastoral systems throughout the African Sahel, providing essential products, including wood, fuel, food, livestock fodder and medicines and enhancing soil fertility (Weber et al. 2008). Seeds are widely dispersed by browsing animals, such as camels, cattle and goats at the end of the dry season (Tybirk 1991) and perhaps also by humans who collect the pods to feed to their animals, and cow dung (containing viable seeds) to fertilise their fields.

#### Etymology.

*Anonychium* literally meaning the absence of nails or claws from the Latin or Greek ‘onych’ = ‘ónyx’ meaning nail or claw, refers to the lack of armature of this genus.

#### Affinities.

*Prosopisafricana* has long been considered anomalous within the genus and was placed in its own section Anonychium by Bentham (1842) and later this was upranked to its own genus, *Anonychium* by Schweinfurth (1868; under the name *A.lanceolatum* Schweinf.). Unlike almost all other species of *Prosopis* s.l., *P.africana* lacks armature, has internally glabrous petals, pollen with costae (Guinet 1969), V-shaped anthers with small stomia forming short pockets on the ventral surface of the anthers and anther glands that are apparently morphologically unique within mimosoids (Luckow and Grimes 1997). The anther glands of *Anonychiumafricanum* (as *P.africana*, Luckow and Grimes 1997: Figs 25–27) stand out as quite different from the typical mimosoid claviform anther glands of the remaining species of *Prosopis* s.l., being sessile, borne ventrally between the thecae, rather than stipitate borne apically or dorsally from the connective between the thecae as in most other mimosoids and forming triangular hood-shaped protrusions made up of papillate cells which are also unique amongst mimosoid anther glands (Luckow and Grimes 1997). Alongside the robust molecular evidence for placement of *P.africana* distantly related to the rest of *Prosopis* (Fig. 1), this suite of morphological differences amply justifies segregation of *P.africana* as a distinct monospecific genus.

*Anonychium* is a phylogenetically isolated lineage that subtends the grade of other unarmed, mainly species-poor genera, *Plathymenia*, *Fillaeopsis* and *Newtonia* which is paraphyletic with respect to the core mimosoid clade of Koenen et al. (2020) (Fig. 1; Ringelberg et al. 2022). This is in line with pollen of *Anonychium* which shows similarities to *Newtonia* (Guinet 1969).

### ﻿ Anonychium africanum

Taxon classificationPlantaeFabalesLeguminosae

(Guill. & Perr.) C.E. Hughes & G.P. Lewis comb. nov.

EAEDDE5E-BD2A-5756-A1F7-F706FACC3973

urn:lsid:ipni.org:names:77303578-1

Prosopis oblonga Benth., J. Bot. (Hooker) 4: 348. 1842.
Entada durissima Baill., Adansonia 6: 208. 1866.
Anonychium lanceolatum Schweinf., Reliq. Kotschy.: 7, pl. 7. 1868.
Prosopis africana (Guill. & Perr.) Taub. in H.G.A. Engler & K.A.E. Prantl, Nat. Pflanzenfam. 3(3): 119. 1892.
Entada coulteri Roberty, Bull. Inst. Fondam. Afrique Noire, Sér. A, Sci. Nat. 16: 346. 1954.

#### Basionym.

*Coulteriaafricana* Guill. & Perr., Fl. Seneg. Tent.: 256, 1832.

#### Type material.

Senegal. Kounoun, Presqu’île du Cap-Vert, *G.S. Perrottet 20* (holotype: P [P00418356]).

### ﻿ Prosopis

Taxon classificationPlantaeFabalesLeguminosae

L., Mantissa Pl. 68: 10. 1767. emend. C.E. Hughes & G.P. Lewis.

1FDCDDFB-D7F2-51D0-A185-5B2AA5DBE797

Lagonychium M. Bieb., Fl. Taur.-Caucas. 3: 288. 1819.
Prosopis section Adenopis DC., Prodr. 2: 446. 1825.
Pleuromenes Raf., Sylva Tellur.: 144. 1838.

#### Type.

*Prosopisspicigera* L., a synonym of *P.cineraria* (L.) Druce.

#### Description.

Prickly subshrubs, shrubs, small trees or occasionally lianescent (*P.farcta*), 0.3–6.5 (–10) m high, deep-rooted and sometimes invading via root suckers, prickles internodal, scattered, straight, somewhat acroscopic, conical with broad bases, 3–5 mm long (Figs 2C, M and 3C), stipular or axillary spines absent. Stipules foliaceous, ovate-acute, caducous. Leaves with 1–6 (–7) pairs of pinnae, the petiole and rachis 0.5–4 cm, sometimes a prickle at the base of the petiole, the pinnular rachises 2–7 cm long, with 7–15 pairs of leaflets, these ovate or lanceolate, straight to sub-falcate or auriculate, mucronate, 2–15 × 2–4.5 mm, glabrous, puberulous or pubescent, mid-vein excentric. Inflorescences spicate, 4–13 cm long, axillary, solitary or in fascicles, peduncle sometimes with an amplexicaul bract, this caducous and leaving an oblique scar; pedicels 0.5–1.5 mm. Flowers small, yellow, yellowish-white, green or cream-green; calyx truncate, 0.8–1.2 mm long; corolla 3.5–4 mm long, the petals linear, nearly free, reflexed, glabrous on both sides; anthers with a minute caducous incurved claviform gland arising from the connective; pollen lacking costae on the pores, tectum irregularly areolate-verrucose. Fruits indehiscent, slender, elongate straight or sub-falcate, dark reddish-brown to blackish, shiny, cylindrical to sub-cylindrical, torulose, 1.5–19 × 0.4–2.5 cm, exocarp thin, brittle, shiny and smooth, orange-red becoming brown, red or black when ripe (Fig. 7C), mesocarp spongy, endocarp segments thin, little developed, seed chambers longitudinal or transverse. Seeds well separated, longitudinal, ovate to ovoid, compressed, 6–8.5 × 5–6 × 2.5–3 mm.

#### Geographic distribution.

Reduced now to just three Old World species, these distributed across arid parts of North Africa (but apparently the genus rare at its western limits in Algeria and Tunisia), the Middle East and NW India (especially Punjab and Rajasthan) and reaching its northern limits in Afghanistan and Azerbaijan (Fig. 8).

#### Habitat and uses.

Abundant in dry and arid parts of NW India, where it is sometimes the most common tree in parts of Punjab and Rajasthan and abundant in arid thorn scrub in parts of the Near East (where *P.farcta*, which can spread via root suckers, is sometimes considered weedy), tolerating saline soils. Highly valued as a source of high quality durable wood, pods for livestock feed and bee forage.

#### Etymology.

Pasiecznick et al. (2001) suggested the name to be derived from *pros*- (Gk.: towards) and *Opis* (wife of Saturn, the Greek goddess of abundance and agriculture), hence ‘towards agriculture’ referring to the widespread utility of the genus.

#### Affinities.

*Prosopis* s.s. is here reduced to three species and is sister to the monospecific genus *Indopiptadenia* (Fig. 1). These two genera share stem/internodal prickles and a W-C Asian distribution that is unique within mimosoids.

### ﻿ Prosopis cineraria

Taxon classificationPlantaeFabalesLeguminosae

(L.) Druce, Rep. Bot. Exch. Club Soc. Brit. Isles 3: 422. 1913. (publ. 1914).

468EC030-50B9-5B3A-9212-1C2C0D1421DC

Mimosa cinerea L., pro parte, Sp. Pl.: 517. 1753 (see note below).
Prosopis spicigera L., Mant. Pl.: 68. 1767.
Prosopis spicata Burm.f., Fl. Indica: 102. 1768.
Prosopis aculeata J. Koenig ex Roxb., Asiat. Res. 4: 405. 1795.
Adenanthera aculeata (J. Koenig ex Roxb.) W. Hunter, Asiat. Res. 6: 66. 1799.
Acacia cineraria (L.) Willd., Sp. Pl., ed. 4, 4: 1057. 1806.

#### Note.

The name *Mimosacineraria* L. (Syst. Nat., ed. 10: 1311. 1759), based on *M.cinerea* L. (Sp. Pl.: 517 [non 520]. 1753; see Art. 53 Ex. 19), was transferred to *Prosopis* L. by Druce (in Bot. Exch. Club Brit. Isles Rep. 3: 422. 1914) as *P.cineraria* (L.) Druce. However, the correct name in *Prosopis* would have been a combination based on *M.cinerea* (l.c.) had not that name been successfully proposed for rejection (see App. V). in ICN Art. 53.5, Note 4.

#### Type material.

India.

### ﻿ Prosopis farcta

Taxon classificationPlantaeFabalesLeguminosae

(Banks & Sol.) J.F. Macbr., Contr. Gray Herb. 59: 17. 1919.

DF50A737-2862-58CF-B8F3-BF23ECD70BAB

Mimosa farcta Banks & Sol. in A. Russell, Nat. Hist. Aleppo, ed. 2, 2: 266. 1794.
Mimosa stephaniana M. Bieb., Tabl. Prov. Mer Casp.: 120. 1798.
Acacia stephaniana (M. Bieb.) Willd., Sp. Pl., ed. 4, 4: 1088. 1806.
Acacia heterocarpa Delile, Descr. Egypte, Hist. Nat.: 79. 1813.
Lagonychium stephanianum (M. Bieb.) M. Bieb., Fl. Taur.-Caucas. 3: 288. 1819.
Mimosa arvensis Sieber ex Steud., Nomencl. Bot. 1: 533. 1821, nom invalid.
Prosopis stephaniana (M. Bieb.) Kunth ex Spreng., Syst. Veg. 2: 326. 1825.
Mimosa agrestis Sieber ex Spreng., Syst. Veg. 2: 206. 1825.
Pleuromenes heterocarpa Raf., Sylva Tellur.: 145. 1838.
Acacia persica Sterler ex Steud., Nomencl. Bot., ed. 2, 1: 7. 1840.
Mimosa micrantha Vahl ex Walp., Repert. Bot. Syst. 5: 582. 1846.
Lagonychium farctum (Banks & Sol.) Bobrov in V.L. Komarov (ed.), Fl. URSS 11: 14. 1941.
Prosopis farcta var. glabra Burkart, J. Arnold Arbor. 57: 454. 1976.

#### Type material.

Syria. Aleppo, without collector; no additional information in protologue.

### ﻿ Prosopis koelziana

Taxon classificationPlantaeFabalesLeguminosae

Burkart, J. Arnold Arbor. 57: 455. 1976.

DA389DC2-E724-539E-A574-ED5C69F4EC62

Prosopis koelziana var. puberula J. Léonard, Bull. Jard. Bot. Natl. Belg. 56: 485. 1986.

#### Type material.

Iran. Madenu, Kirman, *Koelz 14246* (holotype: US [US00000985]).

### ﻿ Strombocarpa

Taxon classificationPlantaeFabalesLeguminosae

(Benth.) Engelm. & A. Gray, Boston J. Nat. Hist. 5: 243. 1845.

4909AF2D-AB6F-5D6F-A8CC-4A7DC3D5E580

Spirolobium A.D. Orb., Voy. Amér. Mér. 8 (Atlas, Bot): t. 13. 1839, nom. rej., non Spirolobium Baill. 1889. (Apocynaceae).
Prosopis sect. Strombocarpa Benth., J. Bot. (Hooker) 4: 351. 1841.
Sopropis Britton & Rose in N.L. Britton & al. (eds.), N. Amer. Fl. 23: 182. 1928.

#### Type.

*Prosopisstrombulifera* (Lam.) Benth. [= *Strombocarpastrombulifera* (Lam.) A. Gray].

#### Description.

Low spiny, sometimes creeping, shrubs or small trees, 0.15–3 (–18) m high, multi-stemmed from the base or sometimes with a short trunk to 10–30 (–45) cm diameter, usually densely and intricately much-branched, some species forming long underground, spreading, horizontal runners (gemmiferous roots or rhizomes), armed with strongly decurrent, straight, cinereous spiny stipules (Figs 2E, H, I and 3A), 0.1–3.5 (–5.5) cm long, brachyblasts congested, blackish. Leaves always unijugate, the petiole (0.5–) 2–15 mm, the pinnular rachises 1–4 cm long, with 3–30 pairs of well separated, alternate to opposite leaflets, these oblong or elliptic-oblong, obtuse to subacute, veins lacking or weakly 1–3-veined, 2–12 × 0.6–4 mm, glaucous, puberulous or glabrescent. Inflorescences axillary, solitary, globose, ovoid-elliptic heads to 1.5 cm diameter at anthesis or shortly cylindrical-spicate, 3–8 cm long. Flowers small, bright or lemon yellow, young filaments red; calyx, 1.5–2.3 mm long; corolla 3–4 (–6) mm long, the petals linear, partially united, villous within; stamens and style exserted, anthers with a minute, caducous, incurved claviform gland arising from the connective. Fruits densely clustered with 1–21 per flower head, indehiscent, lemon-yellow, straw-yellow or reddish-brown when ripe, slender, elongate, straight or falcate (in *S.palmeri* and *S.ferox*; Figs 5C, E and 7E–F), but usually more or less tightly spirally coiled (like corkscrews) with (1–) 8–19 (–24) regular coils, forming a cylindrical body 1.8–5.5 × 0.6–1.5 cm (Figs 5F, G and 7D) or irregularly and more openly coiled; exocarp crustaceous, mesocarp thin or more usually thick and pulpy, tannic, reddish, endocarp delicately segmented in longitudinal or transverse seed chambers which are easy to open or hard and closed. Seeds ovate or reniform ovoid, grey-green, 3–6 (–7) × 3–4 mm.

#### Geographic distribution.

Ten species. Restricted to the New World and there occupying a markedly bicentric amphitropical distribution in arid and semi-arid regions of N. America (southern U.S.A., especially in the Sonoran Desert, Baja California and northern Mexico (Coahuila)) and S. America (south-central Peru to Argentina and Chile) (Fig. 8).

#### Habitat and uses.

In cactus-rich semi-desert Monte vegetation, deserts and arid mesetas, dry river beds and washes and in the hyper-arid Pampa del Tamarugal in northern Chile (*S.tamarugo*), where it is the only tree present and dependent on moisture absorbed from fog. Fruits browsed by cattle and sheep and much valued in arid deserts for that purpose. Wood valued for fuel, and occasionally cultivated (*S.tamarugo*).

#### Etymology.

*Strombo*- (Italian. = conch) and -*carpa* (Gk. = fruit), referring to the resemblance of the fruits to the spiral shells of tropical marine molluscs (see Figs 5F, G and 7D).

#### Affinities.

*Strombocarpa* is robustly supported in recent molecular phylogenies as sister to the African monospecific genus *Xerocladia* (Fig. 1; Ringelberg et al. 2022). These two genera share the diagnostic synapomorphy of stipular spines which are not found elsewhere in *Prosopis* s.l.

### ﻿ Strombocarpa abbreviata

Taxon classificationPlantaeFabalesLeguminosae

(Benth.) Hutch., Gen. Fl. Pl. 1: 287. 1964.

6C178426-A067-5412-B81B-B37E51498965

Prosopis abbreviata Benth., J. Bot. (Hooker) 4: 352. 1842.

#### Type material.

?Argentina. “San Jago”, *Tweedie 168* (holotype: K [K000504799]).

### ﻿ Strombocarpa burkartii

Taxon classificationPlantaeFabalesLeguminosae

(Muñoz) C.E. Hughes & G.P. Lewis comb. nov.

B7DF00D8-ADB3-55FE-BBC0-981B63C3E2D5

urn:lsid:ipni.org:names:77303579-1

#### Basionym.

*Prosopisburkartii* Muñoz, Bol. Mus. Nac. Hist. Nat., Santiago de Chile 32: 364. 1971.

#### Type material.

Chile. Prov. Tarapacá, Pampa del Tamarugal, El Gobierno, sector La Huaica, *C. Muñoz Pizarro 7370* (holotype: SGO [SGO000002436]).

### ﻿ Strombocarpa cinerascens

Taxon classificationPlantaeFabalesLeguminosae

A. Gray, Smithsonian Contr. Knowl. 3(5): 61. 1852.

BA77AE17-71B0-564D-B6E6-4F2627255C73

Prosopis cinerascens (A. Gray) Benth., Trans. Linn. Soc. London 30: 381. 1875.
Prosopis reptans var. cinerascens (A. Gray) Burkart, Darwiniana 4: 75. 1940.
Prosopis reptans subsp. cinerascens (A. Gray) A.E. Murray, Kalmia 13: 24. 1983.
Mimosa calcarea Buckley, Proc. Acad. Nat. Sci. Philadelphia 1861: 453. 1862.

#### Type material.

Mexico. Nuevo León (“New Leon”), valley near Azufrosa, *Gregg 492* (holotype: GH [GH00003469]; isotypes: K [K000791013], MO [MO356342]).

### ﻿ Strombocarpa ferox

Taxon classificationPlantaeFabalesLeguminosae

(Griseb.) C.E. Hughes & G.P. Lewis comb. nov.

7EE4B96F-F55D-5BE0-AEBF-F5E89E654820

urn:lsid:ipni.org:names:77303580-1

#### Basionym.

*Prosopisferox* Griseb., Abh. Königl. Ges. Wiss. Göttingen 24: 118. 1879.

#### Type material.

Argentina. “in regione Puna pr. Humaguaca, pr S José de Tilcara”, Jujuy, Humahuaca, *P.G. Lorentz* & *G.H.E.W. Hieronymus 776* (lectotype: GOET [GOET009646]; isolectotypes: CORD [CORD00004889], F [F0BN001461], SI [SI002480]).

### ﻿ Strombocarpa palmeri

Taxon classificationPlantaeFabalesLeguminosae

(S. Watson) C.E. Hughes & G.P. Lewis comb. nov.

16ABCEAF-0C66-5F01-A0EC-203647CAD5C4

urn:lsid:ipni.org:names:77303581-1

#### Basionym.

*Prosopispalmeri* S. Watson, Proc. Amer. Acad. Arts 24: 48. 1889.

*Sopropispalmeri* (S. Watson) Britton & Rose in N.L. Britton & al. (eds.), N. Amer. Fl. 23: 183. 1928.

#### Type material.

Mexico. Eastern Baja California, Mulegé, *E. Palmer 2* (isotypes: BM [BM000952298], GH [GH00003471], K [K000478262], NDG [NDG24111], NY [NY00005123], US [US00930830]).

### ﻿ Strombocarpa pubescens

Taxon classificationPlantaeFabalesLeguminosae

(Benth.) A. Gray, Smithsonian Contr. Knowl. 3(5): 60. 1852.

E5222871-51A1-5C06-B554-78A0033109D0

Prosopis pubescens Benth., London J. Bot. 5: 82. 1846.
Prosopis emoryi Torr. In W.H. Emory, Not. Milit. Reconn. 2: 189. 1848.
Strombocarpa brevifolia Nutt. ex A. Gray, Smithsonian Contr. Knowl. 3(5): 60. 1852.

#### Type material.

U.S.A. California: between San Miguel and Monterey, *Coulter s.n.*

### ﻿ Strombocarpa reptans

Taxon classificationPlantaeFabalesLeguminosae

(Benth.) A. Gray, U.S. Expl. Exped., Phan. 1: 475. 1854.

AB760793-1103-55D7-9700-733FE4177A20

Prosopis reptans Benth., J. Bot. (Hooker) 4: 352. 1842.
Prosopis abbreviata var. argentina Griseb., Abh. Königl. Ges. Wiss. Göttingen 19: 133. 1874.

#### Type material.

South America. with the label “Mortworta of Cordova, used as a cure for Dysentery” , *Tweedie s.n.* (K [K000504784]).

### ﻿ Strombocarpa strombulifera

Taxon classificationPlantaeFabalesLeguminosae

(Lam.) A. Gray, U.S. Expl. Exped., Phan. 1: 475. 1854.

9193CD42-E0B1-5C1E-B903-294804C10655

Mimosa strombulifera (“strumbulifera”) Lam., Encycl. 1: 15. 1783.
Acacia strombulifera (Lam.) Willd., Sp. Pl., ed. 4, 4: 1055. 1806.
Prosopis strombulifera (Lam.) Benth., J. Bot. (Hooker) 4: 352. 1842.

#### Type material.

Peru. no further details in protologue of *Mimosastrombulifera*.

### ﻿ Strombocarpa strombulifera var. ruiziana

Taxon classificationPlantaeFabalesLeguminosae

(Burkart) C.E. Hughes & G.P. Lewis comb. nov.

3034E257-0F74-53B0-85C6-192F56423D38

urn:lsid:ipni.org:names:77303582-1

#### Basionym.

Prosopisstrombuliferavar.ruiziana Burkart, J. Arnold Arbor. 57: 459. 1976.

#### Type material.

Argentina. Mendoza: Dept. Junín, in aridis salsis inter Barrancas et Rodríguez Peña, *A. Ruiz Leal 3787* (holotype: SI [SI002507]).

### ﻿ Strombocarpa strombulifera var. strombulifera

Taxon classificationPlantaeFabalesLeguminosae

847AD208-3A14-5674-BB36-AB7FA0D3D7B0

Mimosa retortunium Lam., Encycl. 1: 15. 1783, nom. invalid pro syn.
Mimosa circinalis Cav., Icon. 6: 41. 1801, nom. illeg.
Spirolobium australe A.D. Orb., Voy. Amér. Mér. 8 (Atlas, Bot): t. 13. 1839.

### ﻿ Strombocarpa tamarugo

Taxon classificationPlantaeFabalesLeguminosae

(Phil.) C.E. Hughes & G.P. Lewis comb. nov.

7B88FF46-CFE3-5EC2-8BA6-2A4B4F1325E9

urn:lsid:ipni.org:names:77303583-1

#### Basionym.

*Prosopistamarugo* Phil., Anales Mus. Nac. Santiago de Chile 1891: 21. 1891.

#### Type material.

Chile. Prov. Tarapacá, Valle de Tamarugal, *F. Philippi 1840* (holotype: SGO [SGO000002445]; isotype: SI [SI002508]).

### ﻿ Strombocarpa torquata

Taxon classificationPlantaeFabalesLeguminosae

(Lag.) Hutch., Gen. Fl. Pl. 1: 287. 1964.

3177F664-6221-5277-B2F2-035898DC3443

Acacia torquata Lag., Gen. Sp. Pl.: 16, 206. 1816.
Prosopis torquata (Lag.) DC., Prodr. 2: 448. 1825.
Prosopis adesmioides Griseb., Abh. Königl. Ges. Wiss. Göttingen 19: 132. 1874.

#### Type material.

probably t.36, ined., del Hortus de Cavanilles (fide Burkart in Darwiniana 4: 66. 1940).

### ﻿ Neltuma

Taxon classificationPlantaeFabalesLeguminosae

Raf., Sylva Tellur.: 119. 1838.

54E79AEA-C3D6-5F40-B3F1-1507B845768E

Prosopis sect. Algarobia DC. Prodr. 2: 446. 1825.
Mitostax Raf., Sylva Tellur.: 120. 1838.
Algarobia (DC.) Benth., Pl. Hartw.: 13. 1839.
Prosopis sect. Monilicarpa Ruiz Leal ex Burkart, J. Arnold Arbor. 57(3): 230. 1976.

#### Type.

*Neltumajuliflora* (Sw.) Raf. [= *Mimosajuliflora* Sw.].

#### Description.

Spiny, erect to prostrate subshrubs, shrubs and small trees, (0.1–) 4–10 (–20) m high, usually with a short trunk to 40–60 (–>100) cm diameter, branching lax with a spreading rounded or flat-topped crown, twigs cylindrical, flexuous, often arched downwards, glabrous, green or reddish, often with rather long internodes, armed with uninodal axillary, solitary or paired, straight, strong, cylindrical, subulate spines (Figs 2 and 3E), these not necessarily at all nodes, 0.2–15 (–33) cm long × 0.2–1.4 cm in diameter and sometimes thicker than the subtending twig, or with spinescent rigid straight cylindrical branchlets 8–50 cm, brachyblasts congested, blackish. Stipules small, triangular and dry. Leaves with 1–3 (–8) pairs of pinnae, the petiole (0.2–) 2–7.5 cm long, the pinnular rachises (0.2–) 4–19 (–24.5) cm long, with (1–) 2–30 (–50) pairs of opposite leaflets, these linear, ovate-oblong, oblong-linear or lance-ovate, more or less acute, palmately pinnativeined or almost without veins, (0.15–) 2.5–10 × 0.05–3.5 cm, puberulous to scarcely ciliolate or glabrous, or sometimes aphyllous or subaphyllous (*N.sericantha*, *N.kuntzei*), the leaves small and soon falling off the young developing shoots which become spinescent. Inflorescences axillary, solitary or fascicled, spicate, (1.5–) 3–15 cm long with 20–250 flowers on short 1.6 mm pedicels. Flowers white, yellow, greenish-yellow or occasionally red, often perfumed, sometimes some functionally male flowers; calyx 1–2 mm long; corolla 3–5 mm long, the petals almost free, pubescent, usually villous within; stamens and style exserted, anthers with a minute caducous incurved claviform gland arising from the connective. Fruits linear moniliform or compressed turgid (Figs 6 and 7H–I), straw yellow, sometimes tinged reddish-maroon or black, 1–several per infructescence, indehiscent, glabrous, mostly straight to subfalcate, S- or C-shaped or annular with 1–3 very lax open spirals, acuminate, (2–) 5–29 cm in length × 0.5–2.6 cm diameter, margins often thickened and undulate, valves striate corrugate or smooth, exocarp crustaceous, mesocarp thin or more usually thick and pulpy, mealy or spongy, dry, usually sweet, endocarp hard and bony or coriaceous, with convex faces and acute extremities, segmented in longitudinal or transverse subquadrate closed seed chambers. Seeds brown, compressed ovate, 5–10 × 3–6 mm. See also Johnston (1962).

#### Geographic distribution.

Potentially up to 43 species, but probably somewhat fewer (see below). Widespread across seasonally dry tropical and arid regions of the Americas with a pseudo-amphitropical bicentric pattern of greatest species diversity in the Mexican-Texan and Argentinian-Chilean-Paraguayan regions, especially diverse and abundant in the Chaco, with an outlying disjunct occurrence of *Neltumaruscifolia* of questionable nativity in the Caatinga in north-east Brazil (Burkart 1976; Oliveira & Queiroz 2020) and extending into warm and some colder temperate areas in Texas and Nevada in the north and Patagonia in the south, where *N.denudans* Benth. reaches 48 °S (Fig. 8).

#### Habitat and uses.

Dominant across large tracts of the Gran Chaco in mixed sub-xerophyllous woodland, also in Monte vegetation, open desert forests in quebradas along seasonal rivers, in *Stipa*-dominated pampas and semi-desert shrub steppe with hot summers and cold winters in Patagonia as far as 48 °S, some species capable of surviving extreme drought; spanning a wide range of substrates and edaphic conditions including stony and sandy mesas, coastal and inland sand dunes and deep black seasonally inundated, sometimes saline, clay vertisols. Some species weedy and invasive, both within their native ranges and where introduced (see Introduction). The wood generally hard, dense, durable and flexible and widely used for fence posts, parquet flooring, barrels, firewood and charcoal and the fruits are eagerly consumed by all forms of livestock (see Introduction).

#### Etymology.

Possibly derived from the common name *Mulla Thumma* in the Dravidian language Teluga in the Indian states of Andhra Pradesh and Telangana, where *Neltumajuliflora* is introduced.

#### Affinities.

*Neltuma* is sister to, but deeply divergent from, the combined *Strombocarpa* + *Xerocladia* clade (Fig. 1).

Thirteen species of *Prosopis* have been described since the publication of Burkart’s (1976) monograph. One of these, *Prosopisbonplanda* P.R. Earl & Lux, was already placed in synonymy under *P.glandulosa* by Palacios (2006). All of the rest can be confidently placed in *Neltuma* (= Prosopissect.Algarobia + Prosopissect.Monilicarpa), based on morphological descriptions and illustrations from their respective protologues. We provide new combinations in *Neltuma* for all these names, listing potentially up to 43 species for the genus, but we suspect that some of these new species may be no more than regional variants of the widespread and taxonomically difficult *N.pallida* / *N.juliflora* species complex. Given the difficulties of species delimitation across parts of *Neltuma*, we suggest that a detailed molecular study with complete sampling of species and dense sampling of multiple accessions, representing intraspecific diversity, is needed to properly re-assess species boundaries and possible hybridisation. The *Mimobaits* gene set of Koenen et al. (2020) would be an ideal tool for such a study.

### ﻿ Neltuma affinis

Taxon classificationPlantaeFabalesLeguminosae

(Spreng.) C.E. Hughes & G.P. Lewis comb. nov.

8CA4B699-02BC-5E8E-92D4-4CE9B30D9206

urn:lsid:ipni.org:names:77303584-1

Prosopis algarobilla Griseb., Abh. Königl. Ges. Wiss. Göttingen 19: 131. 1874.
Prosopis nandubey Lorentz ex Griseb., Abh. Königl. Ges. Wiss. Göttingen 24: 117. 1879.
Prosopis algarobilla var. nandubay (Lorentz ex Griseb.) Hassl., Repert. Spec. Nov. Regni Veg. 16: 154. 1919.

#### Basionym.

*Prosopisaffinis* Spreng., Syst. Veg. 2: 326. 1825.

#### Type material.

Uruguay. Montevideo, *F. Sello s.n.* (lectotype (designated by Burkart 1976: 491): MO [MO-954306]).

### ﻿ Neltuma alba

Taxon classificationPlantaeFabalesLeguminosae

(Griseb.) C.E. Hughes & G.P. Lewis comb. nov.

D9F9F446-E7EB-5546-93CB-9E23EF21191A

urn:lsid:ipni.org:names:77303585-1

#### Basionym.

*Prosopisalba* Griseb., Abh. Königl. Ges. Wiss. Göttingen 19: 131. 1874.

#### Type material.

Argentina. Córdoba, Estancia Germania, *Lorentz 5* (isotypes: F [F0BN001457], M [M0218675], MPU [MPU016115], SI [SI002458]).

### ﻿ Neltuma alba var. alba

Taxon classificationPlantaeFabalesLeguminosae

BCD2D0FA-F8F7-5E5C-B726-1C791972371A

Prosopis siliquastrum var. longisiliqua Phil., Anales Mus. Nac. Santiago de Chile 1: 20. 1891.
Prosopis atacamensis Phil., Anales Univ. Chile 84: 444. 1893.

### ﻿ Neltuma alba var. panta

Taxon classificationPlantaeFabalesLeguminosae

(Griseb.) C.E. Hughes & G.P. Lewis comb. nov.

3C9AE208-52DA-5B08-978A-0790EEC23A64

urn:lsid:ipni.org:names:77303586-1

Prosopis panta (Griseb.) Hieron., Bol. Acad. Nac. Ci. Republ. Argent. 4: 284. 1881.

#### Basionym.

Prosopisalbavar.panta Griseb., Abh. Königl. Ges. Wiss. Göttingen 24: 118. 1879.

#### Type material.

Argentina. Córdoba, *Lorentz s.n.*

### ﻿ Neltuma alpataco

Taxon classificationPlantaeFabalesLeguminosae

(Phil.) C.E. Hughes & G.P. Lewis comb. nov.

1DE4F7C3-2D14-5125-A5C9-196E37194A7E

urn:lsid:ipni.org:names:77303587-1

#### Basionym.

*Prosopisalpataco* Phil., Anales Univ. Chile 21(2): 394. 1862.

#### Type material.

Argentina. nr Mendoza, *W. Diaz s.n.* (probable isotypes: SGO [SGO000002428], SI [SI002464]).

### ﻿ Neltuma alpataco var. alpataco

Taxon classificationPlantaeFabalesLeguminosae

51C9DEC3-275A-5B4F-863F-1669923689C4

Prosopis stenoloba Phil., Anales Mus. Nac. Santiago de Chile 1: 20. 1891.

### ﻿ Neltuma alpataco var. lamaro

Taxon classificationPlantaeFabalesLeguminosae

(F.A. Roig) C.E. Hughes & G.P. Lewis comb. nov.

2CA1994B-9940-586E-9F07-E08588AA4E38

urn:lsid:ipni.org:names:77303588-1

#### Basionym.

Prosopisalpatacovar.lamaro F.A. Roig, Parodiana 5: 56. 1987. (publ. 1988).

#### Type material.

Argentina. *Roig 8946* (holotype: MERL).

### ﻿ Neltuma alpataco f. rubra

Taxon classificationPlantaeFabalesLeguminosae

(F.A. Roig) C.E. Hughes & G.P. Lewis comb. nov.

E61FCAF4-B16A-5096-A814-DA36970AEEF4

urn:lsid:ipni.org:names:77303589-1

#### Basionym.

Prosopisalpatacof.rubra F.A. Roig, Parodiana 5: 56. 1987. (publ. 1988).

#### Type material.

Argentina. *Roig et al*. *223* (holotype: MERL).

### ﻿ Neltuma andicola

Taxon classificationPlantaeFabalesLeguminosae

(Burkart) C.E. Hughes & G.P. Lewis comb. nov.

C064BD05-1972-5473-B245-14AF5AED7852

urn:lsid:ipni.org:names:77303590-1

Prosopis andicola (Burkart) A. Galán, E. Linares, J. Montoya & Vicente Orell., Phytotaxa 414: 49. 2019.

#### Basionym.

Prosopislaevigatavar.andicola Burkart, J. Arnold Arbor. 57: 510. 1976.

#### Type material.

Peru. Cuzco, Prov. Calca, Hacienda Urco, *J.C. Vargas*-*Calderón 709* (holotype: SI [SI002483]).

### ﻿ Neltuma argentina

Taxon classificationPlantaeFabalesLeguminosae

(Burkart) C.E. Hughes & G.P. Lewis comb. nov.

9C1F8555-AA19-5C3B-A26C-4FA831CDF1E1

urn:lsid:ipni.org:names:77303591-1

#### Basionym.

*Prosopisargentina* Burkart, Revista Argent. Agron. 4: 39. 1937.

#### Type material.

Argentina. Catamarca: Fiambalá, *A. Castellanos s.n.* (holotype: CTES [CTES0000667]; isotype: SI [SI002606]).

### ﻿ Neltuma articulata

Taxon classificationPlantaeFabalesLeguminosae

(S. Watson) Britton & Rose, in N.L. Britton & al. (eds.), N. Amer. Fl. 23: 187. 1928.

8E5F7E0F-E0B6-5D1A-938D-39927A26418E

Prosopis articulata S. Watson, Proc. Amer. Acad. Arts 24: 48. 1889.
Prosopis juliflora var. articulata (S. Watson) Wiggins, Contr. Dudley Herb. 4: 17. 1950.
Neltuma pazensis Britton & Rose, in N.L. Britton & al. (eds.), N. Amer. Fl. 23: 187. 1928.
Prosopis pazensis (Britton & Rose) Wiggins, Contr. Dudley Herb. 4: 18. 1950.

#### Type material.

Mexico. Sonora, Guaymas, *E. Palmer 197* (lectotype designated by Palacios (2006): GH [GH00003478]; isolectotypes: BM [BM000952297, BM000952297], K [K000478261], NY [NY00005127], US [US00000983, US00930831], YU [YU001419]).

### ﻿ Neltuma caldenia

Taxon classificationPlantaeFabalesLeguminosae

(Burkart) C.E. Hughes & G.P. Lewis comb. nov.

C64C5AB1-A446-595F-8CB3-08DA333B8E4E

urn:lsid:ipni.org:names:77303592-1

Prosopis dulcis Gillies ex Hook., Bot. Misc. 3: 203. 1833, nom. illeg.
Prosopis calden Monticelli, Lilloa 3: 348. 1939, nom. nud.

#### Basionym.

*Prosopiscaldenia* Burkart, Darwiniana 3: 111. 1939.

#### Type material.

Argentina. San Luis: Sierra, El Volcán (cerca de la capital), *A.L. Pastore s.n., Herb Burkart 6629* (holotype: SI [SI002466]).

### ﻿ Neltuma calderensis

Taxon classificationPlantaeFabalesLeguminosae

(A. Galán, E. Linares, J. Montoya & Vicente Orell.) C.E. Hughes & G.P. Lewis comb. nov.

637761C5-A112-55A5-8EE7-DF2D01D1F9A2

urn:lsid:ipni.org:names:77303593-1

#### Basionym.

*Prosopiscalderensis* A. Galán, E. Linares, J. Montoya & Vicente Orell., Phytotaxa 414: 50. 2019.

#### Type material.

Peru. Arequipa: Mollebaya, *A. Galán et al*. *AG4633* (holotype: CPUN, isotypes: HUSA, MA, MO, USP).

### ﻿ Neltuma calingastana

Taxon classificationPlantaeFabalesLeguminosae

(Burkart) C.E. Hughes & G.P. Lewis comb. nov.

0B0F9057-9AC8-5DA5-8FC9-9ED530BED948

urn:lsid:ipni.org:names:77303594-1

#### Basionym.

*Prosopiscalingastana* Burkart, Bol. Soc. Argent. Bot. 6: 223. 1957.

#### Type material.

Argentina. San Juan, Calingasta, Quebrada Las Leñas y Est. Las Hornillas, Valle de Los Patos, *Moreau & Perrone s.n.* (*BA55032*) (holotype: SI [SI002468]).

### ﻿ Neltuma campestris

Taxon classificationPlantaeFabalesLeguminosae

(Griseb.) C.E. Hughes & G.P. Lewis comb. nov.

272367D7-92C8-55BE-AA85-E322FFFA23B3

urn:lsid:ipni.org:names:77303595-1

#### Basionym.

*Prosopiscampestris* Griseb., Abh. Königl. Ges. Wiss. Göttingen 19: 132. 1874.

#### Type material.

Argentina. Córdoba, pr. Chañar, *P.G. Lorentz 2* (holotype: GOET [GOET009644]; isotypes: CORD [CORD00005674], F [F0BN001459], SI [SI002469]).

### ﻿ Neltuma castellanosii

Taxon classificationPlantaeFabalesLeguminosae

(Burkart) C.E. Hughes & G.P. Lewis comb. nov.

0FFB5A22-EBB6-5471-9B3A-FF085A79B4D3

urn:lsid:ipni.org:names:77303728-1

#### Basionym.

*Prosopiscastellanosii* Burkart, Darwiniana 5: 66. 1941.

#### Type material.

Argentina. Mendoza: Payún-Matrú, *A. Castellanos 14253* (*BA 36732*) (holotype: SI [SI002471]; isotypes: LIL [LIL000715], GH [GH00063863]).

### ﻿ Neltuma chilensis

Taxon classificationPlantaeFabalesLeguminosae

(Molina) C.E. Hughes & G.P. Lewis comb. nov.

FF618D34-B017-56A4-8A88-CA30B128ACC0

urn:lsid:ipni.org:names:77303729-1

Prosopis chilensis (Molina) Stuntz, U.S.D.A. Bur. Pl. Industr. Invent. Seeds 31: 85. 1914.

#### Basionym.

*Ceratoniachilensis* Molina, Sag. Stor. Nat. Chili: 172. 1782.

#### Type material.

Chile. (no type details given in protologue to *Ceratoniachilensis*).

### ﻿ Neltuma chilensis var. catamarcana

Taxon classificationPlantaeFabalesLeguminosae

(Burkart) C.E. Hughes & G.P. Lewis comb. nov.

C44462A7-8C10-5056-89EE-D8F00760510A

urn:lsid:ipni.org:names:77303730-1

#### Basionym.

Prosopischilensisvar.catamarcana Burkart, J. Arnold Arbor. 57: 497. 1976.

#### Type material.

Argentina. Prov. Catamarca, Dept. Belén, *Ulibarri 581* (holotype: SI [SI002472, SI002473]).

### ﻿ Neltuma chilensis var. chilensis

Taxon classificationPlantaeFabalesLeguminosae

4D8B8F7B-C96A-5085-94FA-15CB33DD31CA

Acacia siliquastrum Cav. ex Lag., Gen. Sp. Pl.: 16. 1816.
Prosopis siliquastrum (Cav. ex Lag.) DC., Prodr. 2: 447. 1825.
Prosopis siliquosa St.-Lag., Ann. Soc. Bot. Lyon 7: 132. 1880, orth. var.
Prosopis schinopoma Stuck., Bull. Acad. Int. Géogr. Bot. 13: 87. 1904.

### ﻿ Neltuma chilensis var. riojana

Taxon classificationPlantaeFabalesLeguminosae

(Burkart) C.E. Hughes & G.P. Lewis comb. nov.

BDB86B25-BCF7-531C-8445-D7526C20F322

urn:lsid:ipni.org:names:77303731-1

#### Basionym.

Prosopischilensisvar.riojana Burkart, Darwiniana 9: 75. 1949.

#### Type material.

Argentina. Prov. de La Rioja: Quebrada de lka Troya, cerca de Jagüel, *A. Burkart 12355* (holotype: SI [SI002474]).

### ﻿ Neltuma denudans

Taxon classificationPlantaeFabalesLeguminosae

(Benth.) C.E. Hughes & G.P. Lewis comb. nov.

7F046ED8-7791-5880-9449-0029978AA8E1

urn:lsid:ipni.org:names:77303732-1

#### Basionym.

*Prosopisdenudans* Benth., J. Bot. (Hooker) 4: 351. 1842.

#### Type material.

Argentina. Patagonia, Santa Cruz, near Puerto Deseado (“Port Desire”), *Middleton s.n.* (holotype: K [K000504789]).

##### Neltumadenudansvar.denudans

### ﻿ Neltuma denudans var. patagonica

Taxon classificationPlantaeFabalesLeguminosae

(Speg.) C.E. Hughes & G.P. Lewis comb. nov.

E5726482-9D0C-55CC-9263-4512856FAEBA

urn:lsid:ipni.org:names:77303733-1

Prosopis denudans var. patagonica (Speg.) Burkart, J. Arnold Arbor. 57: 480. 1976.

#### Basionym.

*Prosopispatagonica* Speg., Revista Fac. Agron. Univ. Nac. La Plata 3: 510. 1897.

#### Type material.

Argentina. Patagonia, “Golfo de San Jorge ”, *C. Spegazzini s.n.*

### ﻿ Neltuma denudans var. stenocarpa

Taxon classificationPlantaeFabalesLeguminosae

(Burkart) C.E. Hughes & G.P. Lewis comb. nov.

E941D139-3646-5DAE-BC3F-372E66EF324D

urn:lsid:ipni.org:names:77303734-1

#### Basionym.

Prosopisdenudansvar.stenocarpa Burkart, Darwiniana 9: 75. 1949.

#### Type material.

Argentina. Gob. del Chubut: Dept. Rawson, south of Trelew, *A. Krapovickas 4367* (isotypes: SI [SI002475, SI002476], BAB [BAB00000476]).

### ﻿ Neltuma elata

Taxon classificationPlantaeFabalesLeguminosae

(Burkart) C.E. Hughes & G.P. Lewis comb. nov.

9149EBB0-89DC-5AA7-95B0-2882B6E6C9B3

urn:lsid:ipni.org:names:77303735-1

Prosopis elata (Burkart) Burkart, Legum. Argent., ed. 2: 544. 1952.

#### Basionym.

Prosopiscampestrisvar.elata Burkart, Darwiniana 4: 112. 1940.

#### Type material.

Paraguay. Chaco, Puesto Buenos Aires, en el sector Pilcomayo, *T. Rojas 8323* (holotype: SI [SI002477]).

### ﻿ Neltuma fiebrigii

Taxon classificationPlantaeFabalesLeguminosae

(Harms) C.E. Hughes & G.P. Lewis comb. nov.

F5C387D7-1E53-5E79-87D1-66459BEF76D7

urn:lsid:ipni.org:names:77303736-1

#### Basionym.

*Prosopisfiebrigii* Harms, Repert. Spec. Nov. Regni Veg. 13: 524. 1915.

#### Type material.

Paraguay. Chaco, *Fiebrig 1254* (isotypes: F [F0BN001462, F0058760F, F0360901F], G [G00400139], K [K000504802], M [M0218669]).

### ﻿ Neltuma flexuosa

Taxon classificationPlantaeFabalesLeguminosae

(DC.) C.E. Hughes & G.P. Lewis comb. nov.

55922754-9473-5D01-B747-AD4904D26101

urn:lsid:ipni.org:names:77303737-1

Acacia flexuosa Lag., Gen. Sp. Pl.: 16 (1816), nom. illeg.

#### Basionym.

*Prosopisflexuosa* DC., Prodr. 2: 447. 1825.

#### Type material.

Chile.

### ﻿ Neltuma flexuosa var. depressa

Taxon classificationPlantaeFabalesLeguminosae

(F.A. Roig) C.E. Hughes & G.P. Lewis comb. nov.

A7B0B4A5-F4DB-549A-A5F9-E01D7D6B3996

urn:lsid:ipni.org:names:77303738-1

Prosopis juliflora f. fruticosa Hauman, Anales Mus. Nac. Hist. Nat. Buenos Aires 24: 391. 1913.
Prosopis alba f. fruticosa (Hauman) Monticelli, Lilloa 3: 347. 1938.

#### Basionym.

Prosopisflexuosavar.depressa F.A. Roig, Parodiana 5: 53. 1987 (publ. 1988).

#### Type material.

Argentina. Mendoza, Depto. Malargüe, Matancilla, *Roig et al*. “*coleción Sierra de Chachahuén 32*” (neotype: MERL).

### ﻿ Neltuma flexuosa var. flexuosa

Taxon classificationPlantaeFabalesLeguminosae

2D1579C3-541C-5757-A2C2-B9FA0D24BF52

Prosopis juliflora f. arborea Hauman, Anales Mus. Nac. Hist. Nat. Buenos Aires 24: 391. 1913.

### ﻿ Neltuma flexuosa var. fruticosa

Taxon classificationPlantaeFabalesLeguminosae

(Meyen) C.E. Hughes & G.P. Lewis

513236F1-1C12-553C-84F6-4ACB40107C61

Prosopis flexuosa var. fruticosa (Meyen) F.A. Roig, Parodiana 5: 53. 1987. (publ. 1988).

#### Basionym.

*Prosopisfruticosa* Meyen, Observ. Bot. 1: 376. 1834.

#### Type material.

Chile. Prov. de Copiapó, *Roig 12536* (holotype: MERL).

### ﻿ Neltuma flexuosa f. subinermis

Taxon classificationPlantaeFabalesLeguminosae

(Burkart) C.E. Hughes & G.P. Lewis comb. nov.

62486024-D0FB-5FC8-A310-76423E15F9EC

urn:lsid:ipni.org:names:77303739-1

#### Basionym.

Prosopisflexuosaf.subinermis Burkart, J. Arnold Arbor. 57: 513. 1976.

#### Type material.

Argentina. San Juan: Calingasta a Barreal, entre La Isla y Sorocayense, *J.H. Hunziker 6451* (holotype: SI).

### ﻿ Neltuma glandulosa

Taxon classificationPlantaeFabalesLeguminosae

(Torr.) Britton & Rose, in N.L. Britton & al. (eds.), N. Amer. Fl. 23: 186 (1928).

7CADDDC5-4EF8-58FE-988D-48E021D41E64

Prosopis glandulosa Torr., Ann. Lyceum Nat. Hist. New York 2: 192. 1827.
Dasiogyna glandulosa (Torr.) Raf., Atlantic J. 1: 146. 1832.
Algarobia glandulosa (Torr.) Torr. & A. Gray, Fl. N. Amer. 1: 399. 1840.
Prosopis juliflora var. glandulosa (Torr.) Cockerell, Bull. New Mexico Agric. Exp. Sta. 15: 58. 1895.
Prosopis chilensis var. glandulosa (Torr.) Standl., Contr. U.S. Natl. Herb. 23: 1658. 1926.

#### Type material.

U.S.A. New Mexico, Union County, Major Long`s Creek (a tributary of the Canadian River (“on the Canadian”), *James s.n.* (holotype: NY [NY00005945]).

### ﻿ Neltuma glandulosa var. glandulosa

Taxon classificationPlantaeFabalesLeguminosae

EFD03559-395B-5413-BA8D-936253A10565

Prosopis juliflora var. constricta Sarg., Trees & Shrubs 2: 249. 1913.
Neltuma constricta (Sarg.) Britton & Rose, in N.L. Britton & al. (eds.), N. Amer. Fl. 23: 186. 1928.
Neltuma neomexicana Britton, in N.L. Britton & al. (eds.), N. Amer. Fl. 23: 186. 1928.
Prosopis bonplanda P.R. Earl & Lux. Publ. Biol. FCB/UANL. Mex. 5 (2): 38. 1991.

### ﻿ Neltuma glandulosa var. prostrata

Taxon classificationPlantaeFabalesLeguminosae

(Burkart) C.E. Hughes & G.P. Lewis comb. nov.

1EFE1482-A8BC-5B69-B0EB-E60E30203C0C

urn:lsid:ipni.org:names:77303740-1

#### Basionym.

Prosopisglandulosavar.prostrata Burkart, J. Arnold Arbor. 57: 516. 1976.

#### Type material.

U.S.A. Texas: Kleberg County, western part of Laureles Division of King Ranch, *M.C. Johnston 54359* (holotype: COLO; isotype SI [SI015053]).

### ﻿ Neltuma hassleri

Taxon classificationPlantaeFabalesLeguminosae

(Harms) C.E. Hughes & G.P. Lewis comb. nov.

950E07F6-4D4E-5FD0-9A60-F034502375E0

urn:lsid:ipni.org:names:77303741-1

#### Basionym.

*Prosopishassleri* Harms, Repert. Spec. Nov. Regni Veg.13: 523. 1915.

#### Type material.

Paraguay. river Pilcomayo, Puerto Tolderia, *T. Rojas 329* (isotypes: A [A00063864], BM [BM000545192], F [F0BN001463, F0360902F], GH, P).

##### Neltumahasslerivar.hassleri

### ﻿ Neltuma hassleri var. nigroides

Taxon classificationPlantaeFabalesLeguminosae

(Burkart) C.E. Hughes & G.P. Lewis comb. nov.

3E9EDC0F-0FF4-531F-8B02-57386AC29A1E

urn:lsid:ipni.org:names:77303742-1

#### Basionym.

Prosopishasslerivar.nigroides Burkart, J. Arnold Arbor. 57: 479. 1976.

#### Type material.

Argentina. Prov. Santa Fe: Dept. General Obligado, Estancia Las Camelias, *A.E. Ragonese 2423* (holotype: SI [SI002481]).

### ﻿ Neltuma humilis

Taxon classificationPlantaeFabalesLeguminosae

(Gillies ex Hook.) C.E. Hughes & G.P. Lewis comb. nov.

9912BB86-335D-5393-B74A-D6F09ED0981E

urn:lsid:ipni.org:names:77303743-1

#### Basionym.

*Prosopishumilis* Gillies ex Hook., Bot. Misc. 3: 204. 1833.

#### Type material.

Argentina. in the Pampas of Buenos Aires (“Ayres”), *J. Gilles s.n.* (holotype: K [K000504787]; isotypes: E [E00158975, E00158976]).

### ﻿ Neltuma juliflora

Taxon classificationPlantaeFabalesLeguminosae

(Sw.) Raf., Sylva Tellur.: 119. 1838.

0ABF2861-B005-500C-A94C-0015B38FADB8

Mimosa juliflora Sw., Prodr. Veg. Ind. Occ.: 85. 1788.
Acacia juliflora (Sw.) Willd., Sp. Pl., ed. 4, 4: 1076. 1806.
Prosopis juliflora (Sw.) DC., Prodr. 2: 447. 1825.
Algarobia juliflora (Sw.) Heynh., Alph. Aufz. Gew.: 18. 1846.
Entada juliflora (Sw.) Roberty, Bull. Inst. Fondam. Afrique Noire, Sér. A, Sci. Nat. 16: 346. 1954.

#### Type material.

Jamaica. *O.P. Swartz s.n.* (S [S-R-3632, S06-5737]).

### ﻿ Neltuma juliflora var. horrida

Taxon classificationPlantaeFabalesLeguminosae

(Kunth) C.E. Hughes & G.P. Lewis comb. nov.

7557A775-3729-5B0F-9A1D-54FB0795C97C

urn:lsid:ipni.org:names:77303744-1

Prosopis juliflora var. horrida (Kunth) Burkart, J. Arnold Arbor. 57: 502. 1976.

#### Basionym.

*Prosopishorrida* Kunth, Mimoses: 106. 1822.

#### Type material.

Peru. “crescit ad radices Andium orientalium, juxta ripam fluminis Amazonum, inter Tomependa(m) et confluentem Chamaya; item prope litus Oceani Pacifici, in arenosis, inter Piura(m) et Lambayeque”, *Humboldt & Bonpland 3603* (isotypes: P [P00679172, P02734496]).

### ﻿ Neltuma juliflora var. juliflora

Taxon classificationPlantaeFabalesLeguminosae

C34638EE-E6B6-527E-9396-312E046B24F5

Mimosa piliflora Sw., Fl. Ind. Occid. 2: 986. 1800.
Mimosa furcata Desf., Tabl. École Bot.: 180. 1804.
Acacia cumanensis Humb. & Bonpl. ex Willd., Sp. Pl., ed. 4, 4: 1058. 1806.
Mimosa salinarum Vahl, Eclog. Amer. 3: 35. 1807.
Acacia diptera Humb. & Bonpl. ex Willd., Enum. Pl.: 1051. 1809.
Mimosa algarrobo Azara, Voy. Amér. Mér. 2: 483. 1809.
Mimosa cumana Poir., in J.B.A.M. de Lamarck, Encycl., Suppl. 1: 65. 1810.
Mimosa levigata Poir., in J.B.A.M. de Lamarck, Encycl., Suppl. 1: 65. 1810.
Mimosa pallida Poir., in J.B.A.M. de Lamarck, Encycl., Suppl. 1: 65. 1810.
Acacia furcata (Desf.) Desv., J. Bot. Agric. 3: 67. 1814.
Acacia falcata Desf., Tabl. École Bot., ed. 2: 207. 1815, nom. illeg.
Mimosa diptera Poir., in J.B.A.M. de Lamarck, Encycl., Suppl. 5: 529. 1817.
Desmanthus salinarum (Vahl) Steud., Nomencl. Bot. 1: 269. 1821.
Prosopis cumanensis Kunth, in F.W.H. von Humboldt, A.J.A. Bonpland & C.S. Kunth, Nov. Gen. Sp. 6: 310. 1824.
Prosopis inermis Kunth, in F.W.H. von Humboldt, A.J.A. Bonpland & C.S. Kunth, Nov. Gen. Sp. 6: 307. 1824.
Acacia salinarum (Vahl) DC., Prodr. 2: 456. 1825.
Prosopis bracteolata DC., Prodr. 2: 447. 1825.
Prosopis domingensis DC., Prodr. 2: 447. 1825.
Mimosa pseudoschinus Terán & Berland., Mem. Comis. Limites: 11. 1832.
Algarobia dulcis Benth., Pl. Hartw.: 13. 1839.
Prosopis dulcis var. domingensis (DC.) Benth., J. Bot. (Hooker) 4: 350. 1842.
Mimosa laevigata Benth., Linnaea 22: 530. 1849, orth. var.
Prosopis vidaliana Náves, Descr. Prosopsisvidaliana: 15. 1877.
Neltuma bakeri Britton & Rose, in N.L. Britton & al. (eds.), N. Amer. Fl. 23: 185. 1928.
Neltuma occidentalis Britton & Rose, in N.L. Britton & al. (eds.), N. Amer. Fl. 23: 185. 1928.
Neltuma pallescens Britton & Rose, in N.L. Britton & al. (eds.), N. Amer. Fl. 23: 185. 1928.
Prosopis juliflora var. inermis (Kunth) Burkart, J. Arnold Arbor. 57: 502. 1976.

### ﻿ Neltuma kuntzei

Taxon classificationPlantaeFabalesLeguminosae

(Harms ex C.E.O. Kuntze) C.E. Hughes & G.P. Lewis comb. nov.

AAD1B8C3-B576-5624-9D75-04D51D53A8A7

urn:lsid:ipni.org:names:77303745-1

Prosopis barba-tigridis Stuck., Comun. Mus. Nac. Buenos Aires 1: 66. 1899.
Prosopis casadensis Penz., Malpighia 12: 408. 1899.

#### Basionym.

*Prosopiskuntzei* Harms ex C.E.O. Kuntze, Revis. Gen. Pl. 3(2): 71. 1898.

#### Type material.

Bolivia. Sierra de Santa Cruz, *O. Kuntze s.n.* (isotypes: F [F0BN001465], NY [NY00003276] , US [US00000986]).

### ﻿ Neltuma laevigata

Taxon classificationPlantaeFabalesLeguminosae

(Humb. & Bonpl. ex Willd.) Britton & Rose, in N.L. Britton & al. (eds.), N. Amer. Fl. 23: 187. 1928.

ED7DA219-E98E-54DD-94A8-2A2816DE9A2A

Acacia laevigata Humb. & Bonpl. ex Willd., Sp. Pl., ed. 4, 4: 1059. 1806.
Prosopis laevigata (Humb. & Bonpl. ex Willd.) M.C. Johnst., Brittonia 14: 78. 1962.
Prosopis dulcis Kunth, Mimoses: 110. 1822.
Acacia tortuosa Billb. ex Beurl., Kongl. Svenska Vetensk. Acad. Handl., n.s., 2: 24. 1856, nom. illeg.
Mimosa rotundata Sessé & Moc., Pl. Nov. Hisp.: 178. 1890.
Neltuma michoacana Britton & Rose, in N.L. Britton & al. (eds.), N. Amer. Fl. 23: 187. 1928.

#### Type material.

Mexico. “in America meridionali”, Morelos, between Huajintlán (“Guasintlan ”) and Puente de Istla, fide Johnston (1962), *Humboldt & Bonpland* (holotype B, microfiche reproduction Herbarium Willdenow Cat. N. 19132 (MO), fide Palacios (2006).

### ﻿ Neltuma limensis

Taxon classificationPlantaeFabalesLeguminosae

(Benth.) C.E. Hughes & G.P. Lewis comb. nov.

2E26A37D-99D0-5AEA-9A8F-99CC04F236A6

urn:lsid:ipni.org:names:77303746-1

#### Basionym.

*Prosopislimensis* Benth., J. Bot. (Hooker) 4: 350. 1842.

#### Type material.

Peru. Lima, *H. Cuming 974* (lectotype designated by Perry 1998. Fl. Australia 12: 193; isolectotypes: BM [BM000952294], E [E00319916, E00319926], GH [GH00063865], K [K000821140], US).

### ﻿ Neltuma mantaroensis

Taxon classificationPlantaeFabalesLeguminosae

(L. Vásquez, Escurra & Huamán) C.E. Hughes & G.P. Lewis comb. nov.

7E019006-3A69-5A19-B681-462827895BCA

urn:lsid:ipni.org:names:77303747-1

#### Basionym.

*Prosopismantaroensis* L. Vásquez, Escurra & Huamán, Sciéndo 12(1): 70. 2009.

#### Type material.

Peru. Ayacucho, Prov. Huanta, Distr. Huanta, *L. Vásquez Núñez et al*. *12845* (holotype: PRG; isotype: PRG).

### ﻿ Neltuma mayana

Taxon classificationPlantaeFabalesLeguminosae

(R.A. Palacios) C.E. Hughes & G.P. Lewis comb. nov.

F7338200-F748-5090-866C-BB3B3993B787

urn:lsid:ipni.org:names:77303748-1

#### Basionym.

*Prosopismayana* R.A. Palacios, Bol. Soc. Argent. Bot. 41: 115. 2006.

#### Type material.

Mexico. Yucatán, entre Dzilam de Bravo y El Tajo, *R. Palacios 2362* (holotype: MEXU [MEXU01241933]; isotypes: BAFC, TEX [TEX00202236]).

### ﻿ Neltuma mezcalana

Taxon classificationPlantaeFabalesLeguminosae

(R.A. Palacios) C.E. Hughes & G.P. Lewis comb. nov.

7BFA7DF8-4811-59E9-B347-514ACE38AD6B

urn:lsid:ipni.org:names:77303749-1

#### Basionym.

*Prosopismezcalana* R.A. Palacios, Bol. Soc. Argent. Bot. 41: 105. 2006.

#### Type material.

Mexico. Guerrero, entrada a Chacamerito y Tanganhuato, *R. Palacios 2402* (holotype: MEXU; isotypes: BAFC, TEX [TEX00202211]).

### ﻿ Neltuma nigra

Taxon classificationPlantaeFabalesLeguminosae

(Griseb.) C.E. Hughes & G.P. Lewis comb. nov.

FC803235-E5DB-54DF-A839-7913C62F9A84

urn:lsid:ipni.org:names:77303750-1

Prosopis nigra (Griseb.) Hieron., Bol. Acad. Nac. Ci. Republ. Argent. 4: 283. 1881.

#### Basionym.

Prosopisalgarobillavar.nigra Griseb., Abh. Königl. Ges. Wiss. Göttingen 24: 118. 1879.

#### Type material.

Argentina. Córdoba, prope urban Chacra de la Merced, *C. Galander s.n.* (?holotype: HBG [HBG519250].

### ﻿ Neltuma nigra var. longispina

Taxon classificationPlantaeFabalesLeguminosae

(Burkart) C.E. Hughes & G.P. Lewis comb. nov.

F4709515-962E-5702-8E73-754214314458

urn:lsid:ipni.org:names:77303751-1

#### Basionym.

Prosopisnigravar.longispina Burkart, J. Arnold Arbor. 57: 507. 1976.

#### Type material.

Argentina. Prov. Corrientes, Dept. Capital, 2 km S of Paso Pessoa, *T.M. Pedersen 2808* (holotype: SI [SI002485]; isotypes: C [C10012323], CTES [CTES0000668], L [L0019214], MO [MO-954304], WAG [WAG0132133]).

### ﻿ Neltuma nigra var. nigra

Taxon classificationPlantaeFabalesLeguminosae

4D43A2EB-163A-5C0E-9E0B-8024185A525E

Prosopis dulcis var. australis Benth., J. Bot. (Hooker) 4: 350. 1842.

### ﻿ Neltuma nigra var. ragonesei

Taxon classificationPlantaeFabalesLeguminosae

(Burkart) C.E. Hughes & G.P. Lewis comb. nov.

F12F13C6-A707-5B4C-9615-21935686EC5B

urn:lsid:ipni.org:names:77303752-1

#### Basionym.

Prosopisnigravar.ragonesei Burkart, Darwiniana 7: 518. 1947.

#### Type material.

Argentina. Santa Fe: Videla, *A.E. Ragonese 2078* (holotype: SI [SI002490]).

### ﻿ Neltuma nuda

Taxon classificationPlantaeFabalesLeguminosae

(Schinini) C.E. Hughes & G.P. Lewis comb. nov.

EEF88255-FCF2-5A81-8FD9-22293B724C5F

urn:lsid:ipni.org:names:77303753-1

#### Basionym.

*Prosopisnuda* Schinini, Bonplandia (Corrientes) 5: 105. 1981.

#### Type material.

Paraguay. Dep. Boquerón. Mariscal Estigarribia, *A. Schinini* & *E.E. Bordas 15222* (holotype: CTES [CTES0000670]; isotype: SI [SI002493]).

### ﻿ Neltuma odorata

Taxon classificationPlantaeFabalesLeguminosae

(Torr. & Frém.) C.E. Hughes & G.P. Lewis comb. nov.

1E3C1B5A-4269-5B40-9D80-65118733B0B2

urn:lsid:ipni.org:names:77303754-1

Strombocarpa odorata (Torr. & Frém.) A. Gray, U.S. Expl. Exped., Phan. 1: 475. 1854.
Prosopis juliflora var. torreyana L.D. Benson, Amer. J. Bot. 28: 751. 1941.
Prosopis glandulosa var. torreyana (L.D. Benson) M.C. Johnst., Brittonia 14: 82. 1962.
Prosopis glandulosa subsp. torreyana (L.D. Benson) A.E. Murray, Kalmia 12: 23. 1982.

#### Basionym.

*Prosopisodorata* Torr. & Frém., in J.C. Frémont, Rep. Exped. Rocky Mts.: 313. Pl. 1. 1845. Pro parte, excluding the fruits, fide L. D. Benson Madroño 15: 53. 1959.

#### Type material.

U.S.A. California, along Mohave and Virgin River, *Fremont s.n.* (lectotype designated Benson (1959): NY), excluding the fruits.

### ﻿ Neltuma pallida

Taxon classificationPlantaeFabalesLeguminosae

(Humb. & Bonpl. ex Willd.) C.E. Hughes & G.P. Lewis comb. nov.

21B118C0-4DEC-5838-9E34-E8DBBEE738D8

urn:lsid:ipni.org:names:77303755-1

Prosopis pallida (Humb. & Bonpl. ex Willd.) Kunth, in F.W.H. von Humboldt, A.J.A. Bonpland & C.S. Kunth, Nov. Gen. Sp. 6: 309. 1824.
Mitostax pallida (Humb. & Bonpl. ex Willd.) Raf., Sylva Tellur.: 120. 1838.

#### Basionym.

*Acaciapallida* Humb. & Bonpl. ex Willd., Sp. Pl., ed. 4, 4: 1059. 1806.

#### Type material.

Peru. Prov. Jaén de Bracamoros, Passo de Matara, “in America meridionali”, ?Bonpland.

### ﻿ Neltuma palmeri

Taxon classificationPlantaeFabalesLeguminosae

Britton & Rose, in N.L. Britton & al. (eds.), N. Amer. Fl. 23: 185. 1928.

4C2D171F-E805-550B-93E4-22FBF49804B5

Prosopis tamaulipana Burkart, J. Arnold Arbor. 57: 494. 1976.

#### Type material.

Mexico. Tamaulipas: vicinity of Victoria, *E. Palmer 400* (holotype: NY [NY00005077]; isotypes: CM [CM1060], GH, MO [MO-356247], US [US00000993]).

Although the nom. nov. *P.tamaulipana* Burkart was required when *Neltumapalmeri* Britton & Rose was transferred to *Prosopis* because the name *Prosopispalmeri* S. Watson (= *Strombocarpapalmeri* (S.Watson) C.E. Hughes & G.P. Lewis) was already occupied, the original *N.palmeri* provides a valid accepted name.

### ﻿ Neltuma peruviana

Taxon classificationPlantaeFabalesLeguminosae

(L. Vásquez, Escurra & Huamán) C.E. Hughes & G.P. Lewis comb. nov.

7D609A1F-4F65-5DF6-8FF0-ADEB9619F708

urn:lsid:ipni.org:names:77303756-1

#### Basionym.

*Prosopisperuviana* L. Vásquez, Escurra & Huamán, Sciéndo 12(1): 74. 2009.

#### Type material.

Peru. Apurímac, Prov. Andahuaylas, Distr. Sapichaca, *L. Vásquez Núñez et al*. *12849* (holotype: PRG; isotype: PRG).

### ﻿ Neltuma piurensis

Taxon classificationPlantaeFabalesLeguminosae

(L. Vásquez, Escurra & Huamán) C.E. Hughes & G.P. Lewis comb. nov.

BEA71F7A-71AA-58BA-B364-33990678D2E8

urn:lsid:ipni.org:names:77303757-1

#### Basionym.

*Prosopispiurensis* L. Vásquez, Escurra & Huamán, Sciéndo 12(1): 76. 2009.

#### Type material.

Peru. Piura, Prov. Sullana, borde de carretera panamericana cerca al Puente del rio Chira, *L. Vásquez Núñez et al*. *13258* (holotype: PRG).

### ﻿ Neltuma pugionata

Taxon classificationPlantaeFabalesLeguminosae

(Burkart) C.E. Hughes & G.P. Lewis comb. nov.

05242E6C-3CD7-5C37-80EA-EE53FB4DF5C9

urn:lsid:ipni.org:names:77303758-1

#### Basionym.

*Prosopispugionata* Burkart, Darwiniana 9: 70. 1949.

#### Type material.

Argentina. Prov. de Córdoba, extremo noroeste, bosques xerófilos a las Salinas Grandes, km 907, *A.E. Ragonese & B. Piccinini 6097* (holotype: BAB [BAB00000478]; isotype: SI [SI002497]).

### ﻿ Neltuma purpurea

Taxon classificationPlantaeFabalesLeguminosae

(L. Vásquez, Escurra & Huamán) C.E. Hughes & G.P. Lewis comb. nov.

C0EC5CE4-461C-5AC6-AF02-C2E18A3E1EAB

urn:lsid:ipni.org:names:77303759-1

#### Basionym.

*Prosopispurpurea* L. Vásquez, Escurra & Huamán, Sciéndo 12(1): 79. 2009.

#### Type material.

Peru. Tumbes, Distr. Puerto Pizarro, *L. Vásquez Núñez et al*. *12941* (holotype: PRG; isotype: PRG).

### ﻿ Neltuma rojasiana

Taxon classificationPlantaeFabalesLeguminosae

(Burkart) C.E. Hughes & G.P. Lewis comb. nov.

05935AA4-C9B8-5AC3-B40D-08DD9CE9F932

urn:lsid:ipni.org:names:77303760-1

#### Basionym.

*Prosopisrojasiana* Burkart, Darwiniana 5: 70. 1941.

#### Type material.

Paraguay. Chaco paraguayo, Sector López de Filippis, *Rojas 8310* (holotype: SI [SI002500]).

### ﻿ Neltuma rubriflora

Taxon classificationPlantaeFabalesLeguminosae

(Hassl.) C.E. Hughes & G.P. Lewis comb. nov.

ECDBC710-1338-56E8-8F56-3A7EE7C1638A

urn:lsid:ipni.org:names:77303761-1

#### Basionym.

*Prosopisrubriflora* Hassl., Repert. Spec. Nov. Regni Veg. 8: 552. 1910.

#### Type material.

Paraguay. Centurión, zwischen Apa und Aquidaban, *K. Fiebrig 5348* (isotypes: F [F0BN001468], GH [GH00063869], HBG [HBG519244], M [M0218666], P [P02436145], fragment SI [SI002502]).

### ﻿ Neltuma ruizlealii

Taxon classificationPlantaeFabalesLeguminosae

(Burkart) C.E. Hughes & G.P. Lewis comb. nov.

CC3CCFE7-652E-549B-9CC0-4D025AA6A254

urn:lsid:ipni.org:names:77303762-1

#### Basionym.

*Prosopisruizlealii* Burkart, Darwiniana 4: 328. 1942.

#### Type material.

Argentina. Prov. Mendoza, Dep. San Rafael: Agua del Sapo, *Ruiz Leal 7358* (holotype: SI).

### ﻿ Neltuma ruscifolia

Taxon classificationPlantaeFabalesLeguminosae

(Griseb.) C.E. Hughes & G.P. Lewis comb. nov.

BCC5B787-3E84-53AB-806E-DBA0AD74C824

urn:lsid:ipni.org:names:77303763-1

#### Basionym.

*Prosopisruscifolia* Griseb., Abh. Königl. Ges. Wiss. Göttingen 19: 130. 1874.

#### Type material.

Argentina. Santiago del Estero, *P.G. Lorentz 21* (holotype: GOET [GOET009549]; isotypes: CORD [CORD00005670], SI [SI002504]).

### ﻿ Neltuma sericantha

Taxon classificationPlantaeFabalesLeguminosae

(Gillies ex Hook.) C.E. Hughes & G.P. Lewis comb. nov.

0743457F-FA0B-5715-90A3-E4C5E17FE9E2

urn:lsid:ipni.org:names:77303764-1

#### Basionym.

*Prosopissericantha* Gillies ex Hook., Bot. Misc. 3: 204. 1833.

#### Type material.

Argentina. Prov. San Luis, *J. Gilles s.n.* (holotype: K [K000504780]; isotypes: E [E00180081, E00180082], GH [GH00063870]).

### ﻿ Neltuma tupayachensis

Taxon classificationPlantaeFabalesLeguminosae

(L. Vásquez, Escurra & Huamán) C.E. Hughes & G.P. Lewis comb. nov.

F2291886-C022-5BFF-A2D2-87F007A63C90

urn:lsid:ipni.org:names:77303765-1

#### Basionym.

*Prosopistupayachensis* L. Vásquez, Escurra & Huamán, Sciéndo 12(1): 82. 2009.

#### Type material.

Peru. Prov. Cuzco, Distr. Lucre, *L. Vásquez Núñez et al*. *12846* (holotype: PRG; isotype: PRG).

### ﻿ Neltuma velutina

Taxon classificationPlantaeFabalesLeguminosae

(Wooton) Britton & Rose, in N.L. Britton & al. (eds.), N. Amer. Fl. 23: 186. 1928.

247ED5E2-9CA5-5BE5-BCAE-DF0668364AE9

Prosopis velutina Wooton, Bull. Torrey Bot. Club 25: 456. 1898.
Prosopis juliflora var. velutina (Wooton) Sarg., Silva N. Amer. 13: 15. 1902.
Prosopis chilensis var. velutina (Wooton) Standl., Contr. U.S. Natl. Herb. 23: 1658. 1926.

#### Type material.

U.S.A. Arizona, without further locality, *Pringle 13665* (lectotype NY [NY00003272] designated by Britton & Rose in N. Am. Fl. 23(3): 186. 1928; isolectotypes: A [A00003470], CM [CM1091], MO [MO-954307]).

### ﻿ Neltuma × vinalillo

Taxon classificationPlantaeFabalesLeguminosae

(Stuck.) C.E. Hughes & G.P. Lewis comb. nov.

74C82CF3-9B77-5867-B7DF-94EC73A3C7AA

urn:lsid:ipni.org:names:77303766-1

N. alba var. panta (as P.panta)× N.ruscifolia.

#### Basionym.

*Prosopis×vinalillo* Stuck., Anales Mus. Nac. Buenos Aires 7 (ser. 2, t. 4): 73. 1902.

#### Type material.

Argentina. Prov. Tucumán: Depto. De Burruyaco, ? Cañada Alegre.

### ﻿ Neltuma yaquiana

Taxon classificationPlantaeFabalesLeguminosae

(R.A. Palacios) C.E. Hughes & G.P. Lewis comb. nov.

238F765D-E706-52F9-BA18-0FFDE3504478

urn:lsid:ipni.org:names:77303767-1

#### Basionym.

*Prosopisyaquiana* R.A. Palacios, Bol. Soc. Argent. Bot. 41: 117. 2006.

#### Type material.

Mexico. Sinaloa, alrededores del Cementerio de Topolobampo, *R. Palacios 2417* (holotype: MEXU; isotypes: BAFC, TEX [TEX00202225]).

## Supplementary Material

XML Treatment for Anonychium

XML Treatment for Anonychium africanum

XML Treatment for Prosopis

XML Treatment for Prosopis cineraria

XML Treatment for Prosopis farcta

XML Treatment for Prosopis koelziana

XML Treatment for Strombocarpa

XML Treatment for Strombocarpa abbreviata

XML Treatment for Strombocarpa burkartii

XML Treatment for Strombocarpa cinerascens

XML Treatment for Strombocarpa ferox

XML Treatment for Strombocarpa palmeri

XML Treatment for Strombocarpa pubescens

XML Treatment for Strombocarpa reptans

XML Treatment for Strombocarpa strombulifera

XML Treatment for Strombocarpa strombulifera var. ruiziana

XML Treatment for Strombocarpa strombulifera var. strombulifera

XML Treatment for Strombocarpa tamarugo

XML Treatment for Strombocarpa torquata

XML Treatment for Neltuma

XML Treatment for Neltuma affinis

XML Treatment for Neltuma alba

XML Treatment for Neltuma alba var. alba

XML Treatment for Neltuma alba var. panta

XML Treatment for Neltuma alpataco

XML Treatment for Neltuma alpataco var. alpataco

XML Treatment for Neltuma alpataco var. lamaro

XML Treatment for Neltuma alpataco f. rubra

XML Treatment for Neltuma andicola

XML Treatment for Neltuma argentina

XML Treatment for Neltuma articulata

XML Treatment for Neltuma caldenia

XML Treatment for Neltuma calderensis

XML Treatment for Neltuma calingastana

XML Treatment for Neltuma campestris

XML Treatment for Neltuma castellanosii

XML Treatment for Neltuma chilensis

XML Treatment for Neltuma chilensis var. catamarcana

XML Treatment for Neltuma chilensis var. chilensis

XML Treatment for Neltuma chilensis var. riojana

XML Treatment for Neltuma denudans

XML Treatment for Neltuma denudans var. patagonica

XML Treatment for Neltuma denudans var. stenocarpa

XML Treatment for Neltuma elata

XML Treatment for Neltuma fiebrigii

XML Treatment for Neltuma flexuosa

XML Treatment for Neltuma flexuosa var. depressa

XML Treatment for Neltuma flexuosa var. flexuosa

XML Treatment for Neltuma flexuosa var. fruticosa

XML Treatment for Neltuma flexuosa f. subinermis

XML Treatment for Neltuma glandulosa

XML Treatment for Neltuma glandulosa var. glandulosa

XML Treatment for Neltuma glandulosa var. prostrata

XML Treatment for Neltuma hassleri

XML Treatment for Neltuma hassleri var. nigroides

XML Treatment for Neltuma humilis

XML Treatment for Neltuma juliflora

XML Treatment for Neltuma juliflora var. horrida

XML Treatment for Neltuma juliflora var. juliflora

XML Treatment for Neltuma kuntzei

XML Treatment for Neltuma laevigata

XML Treatment for Neltuma limensis

XML Treatment for Neltuma mantaroensis

XML Treatment for Neltuma mayana

XML Treatment for Neltuma mezcalana

XML Treatment for Neltuma nigra

XML Treatment for Neltuma nigra var. longispina

XML Treatment for Neltuma nigra var. nigra

XML Treatment for Neltuma nigra var. ragonesei

XML Treatment for Neltuma nuda

XML Treatment for Neltuma odorata

XML Treatment for Neltuma pallida

XML Treatment for Neltuma palmeri

XML Treatment for Neltuma peruviana

XML Treatment for Neltuma piurensis

XML Treatment for Neltuma pugionata

XML Treatment for Neltuma purpurea

XML Treatment for Neltuma rojasiana

XML Treatment for Neltuma rubriflora

XML Treatment for Neltuma ruizlealii

XML Treatment for Neltuma ruscifolia

XML Treatment for Neltuma sericantha

XML Treatment for Neltuma tupayachensis

XML Treatment for Neltuma velutina

XML Treatment for Neltuma × vinalillo

XML Treatment for Neltuma yaquiana
